# Aberrant expression of miR-133a in endothelial cells inhibits angiogenesis by reducing pro-angiogenic but increasing anti-angiogenic gene expression

**DOI:** 10.1038/s41598-022-19172-x

**Published:** 2022-08-30

**Authors:** Suhail Ahmed, Sathishkumar Kurusamy, Ezra Leander Santhosh David, Kinza Khan, Krithika Kalyanakrishnan, Miebaka Ian-Gobo, Teja Manidhar Kola, Robert N. Wilkinson, Vinodh Kannappan, Weiguang Wang, Manuel J. Gómez, Juan Miguel Redondo, James Cotton, Angel L. Armesilla

**Affiliations:** 1 Cardiovascular Molecular Pharmacology Laboratory, Research Institute in Healthcare Science, School of Pharmacy, Faculty of Science and Engineering, University of Wolverhampton, Wulfruna Street, Wolverhampton, WV1 1SB UK; 2 School of Life Sciences, Medical School, Queens Medical Centre, University of Nottingham, Nottingham, UK; 3 Experimental Cancer Therapeutics Group, Research Institute in Healthcare Science, Faculty of Science and Engineering, University of Wolverhampton, Wolverhampton, UK; 4 Unidad de Bioinformática, Centro Nacional de Investigaciones Cardiovasculares, Madrid, Spain; 5 Gene Regulation in Cardiovascular Remodelling and Inflammation Group, Centro Nacional de Investigaciones Cardiovasculares, Madrid, Spain; 6 Centro de Investigación Biomédica en Red de Enfermedades Cardiovasculares (CIBERCV), Madrid, Spain; 7 Department of Cardiology, Heart and Lung Centre, New Cross Hospital, Wolverhampton, UK; 8 Present Address: Centre for Molecular Processing and School of Biosciences, University of Kent, Canterbury, Kent, CT2 7NJ UK

**Keywords:** Molecular biology, Molecular medicine

## Abstract

Angiogenesis is a multi-factorial physiological process deregulated in human diseases characterised by excessive or insufficient blood vessel formation. Emerging evidence highlights a novel role for microRNAs as regulators of angiogenesis. Previous studies addressing the effect of miR-133a expression in endothelial cells during blood vessel formation have reported conflicting results. Here, we have assessed the specific effect of mature miR-133a strands in angiogenesis and the expression of endothelial angiogenic genes. Transfection of miR-133a-3p or -5p mimics in primary human endothelial cells significantly inhibited proliferation, migration, and tubular morphogenesis of transfected cells. Screening of gene arrays related to angiogenic processes, and further validation by TaqMan qPCR, revealed that aberrant expression of miR-133a-3p led to a decrease in the expression of genes encoding pro-angiogenic molecules, whilst increasing those with anti-angiogenic functions. Ingenuity Pathway Analysis of a collection of genes differentially expressed in cells harbouring miR-133a-3p, predicted decreased cellular functions related to vasculature branching and cell cycle progression, underlining the inhibitory role of miR-133a-3p in angiogenic cellular processes. Our results suggest that controlled delivery of miR-133a-3p mimics, or antagomirs in diseased endothelial cells, might open new therapeutic interventions to treat patients suffering from cardiovascular pathologies that occur with excessive or insufficient angiogenesis.

## Introduction

Angiogenesis, the formation of blood vessels from pre-existing ones, is a multi-factorial physiological process tightly regulated by a balance in the expression of pro- and anti-angiogenic signals^1^. Deregulation of this balance is associated with human diseases characterised by excessive or insufficient blood vessel formation^2^. Among all pro-angiogenic factors identified so far, vascular endothelial growth factor-A_165_ (VEGF-A_165_) stands out as a major inducer of angiogenesis. VEGF-A_165_ exerts its biological function by binding to specific tyrosine kinase receptors located on the surface of endothelial cells. The axis formed by VEGF-A_165_ and its receptor VEGFR2 has been described as a pivotal inducer of both physiological and pathological angiogenesis^3^, although the intracellular regulators that modulate VEGF-A_165_-induced signalling in endothelial cells have not yet been fully characterised. Emerging evidence has recently highlighted a novel role for microRNAs as key cytoplasmic regulators of angiogenesis^4,5^.

microRNAs (miRNAs) are small (~ 22 nucleotides), non-coding, single strand RNA molecules that regulate gene expression post-transcriptionally. In most cases, miRNAs bind to specific sequences located in the 3’UTR of RNAs, inducing their degradation or inhibiting their translation into proteins^6^. Whereas some miRNAs are expressed ubiquitously, others present a tissue-restrictive pattern of expression^7^. This is the case for miR-133a, one of the so-called myo-miRNAs, that is expressed preferentially in muscle-tissue^8,9^. Accordingly, endothelial cells express very low levels of miR-133a under physiological conditions^10^. Recent reports have shown, however, that miR-133a expression is strongly enhanced in the diseased endothelium^10,11^. Previous studies on the effect of ectopic miR-133a in endothelial cells linked it to inhibition of angiogenesis^11,12^. Furthermore, miR-133a antagonism was reported to improve reperfusion of ischaemic limbs in diabetic mice^11^, highlighting the potential therapeutic applications of modulating miR-133a expression in endothelial cells. However, Zhu et al. (2021) recently reported an opposite role for miR-133a as a positive regulator of endothelial cell angiogenesis^13^.

miRNA biogenesis generates two different mature forms, -3p and -5p, depending on the strand of the precursor-miRNA incorporated into the RNA Induced Silencing Complex (RISC) during the strand selection process. In general, both strands can modulate gene expression^14^. Bioinformatic target gene prediction has shown that each miR-133a strand is expected to target different genes, although some genes might still be targeted by both -3p and -5p^15^. Expression of both miR-133a strands has been reported to increase in cardiovascular pathological settings^15–17^. However, the specific effect of the -3p and -5p strand on angiogenesis has not been addressed so far.

To resolve the current disagreement of the role of miR-133a in angiogenesis and to elucidate the molecular and cellular mechanisms regulated by each strand, we have analysed the functional consequences of specific expression of each strand on the regulation of endothelial angiogenic processes. Furthermore, we have determined the changes elicited by each miR-133a strand on the expression of genes encoding key regulators of angiogenesis in primary endothelial cells.

We show that individual expression of each miR-133a strand attenuates tubular morphogenesis, proliferation and migration of human endothelial cells stimulated with VEGF-A_165_, with the effect of the -3p strand being more profound than the inhibition mediated by -5p. Both strands exert their effect by decreasing the expression of angiogenic positive regulators, whilst enhancing expression of angiogenic inhibitors. Although our results show that several genes were commonly targeted by both strands, independent expression of -3p or -5p specifically targeted the expression of different angiogenic regulators. Our results suggest that the modulation of miR-133a (particularly the -3p strand) in diseased endothelial cells might have important therapeutic applications to treat human diseases that occur with angiogenesis.

## Results

### miRNA-133a strands 3p and 5p differentially inhibit VEGF-A_165_-induced angiogenesis

**Figure 1:**
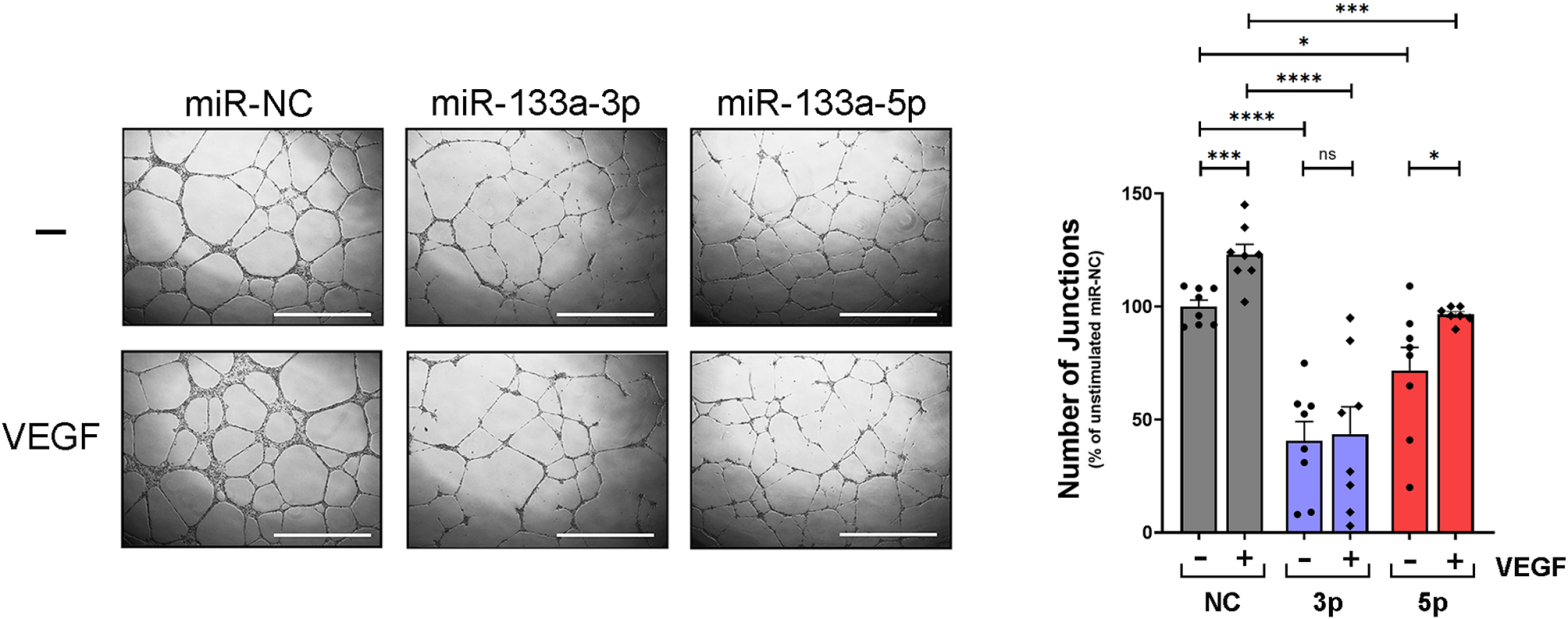
Ectopic expression of miR-133a strands -3p or -5p inhibits endothelial cell tubular morphogenesis. HUVEC transfected with miRNA “mimics” for strand -3p (miR-133a-3p), -5p (miR-133a-5p), or a negative control (miR-NC) were plated on Growth-Factor Reduced Matrigel (Geltrex) in ECGM medium containing 1% FBS and were left untreated (−) or stimulated with VEGF-A_165_ at 50 ng/ml final concentration (+) as indicated. Images show representative fields from experiments quantified in the histogram. Grey, blue and red bars indicate cells transfected with miR-NC, miR-133a-3p and miR-133a-5p respectively. Data are shown as mean ± SE, n = 7. ns = non-significant; *, *P* ≤ 0.05; ***, *P* ≤ 0.001; ****, *P* ≤ 0.0001 (unpaired two-tailed Student’s *t*-test when comparing the indicated groups). Scale bars, 1000 µm. ImageJ (https://imagej.nih.gov/ij/) angiogenesis analyzer plugin software was used to quantify “Number of Junctions” in Matrigel assays.

Previous studies on the effect of aberrant miR-133a expression on endothelial cell angiogenesis have reported conflicting results^11–13^. As a first step to address this issue, we analysed the tubular morphogenesis capabilities of primary endothelial cells specifically overexpressing miR-133a-3p or -5p strands. Ectopic expression of miR-133a-3p strongly inhibited tubular morphogenesis of HUVEC compared to that observed in cells transfected with a control miRNA mimic (miR-NC) (Fig. 1). Furthermore, the presence of miR-133a-3p completely blunted the increase in tube formation induced by VEGF-A_165_ stimulation in control cells (Fig. 1). Overexpression of miR-133a-5p also led to a statistically significant reduction of tubular morphogenesis (Fig. 1), although the effect of this strand was weaker than that observed for 3p. Moreover, tube formation was still significantly increased by VEGF-A_165_ stimulation in cells overexpressing miR-133a-5p (Fig. 1).

To substantiate these results in endothelial cells coming from a different vascular bed, we transfected primary human cardiac microvascular endothelial cells with miRNA mimic control or miR-133a-3p and analysed the ability of the cells to form tubular-like structures in matrigel assays. Consistent with the results observed in HUVEC, miR-133a-3p impaired basal and VEGF-A_165_-induced tubular morphogenesis in microvascular cardiac endothelial cells (Fig. S1A). Likewise, transfection of miR-133a-3p in primary human aortic endothelial cells also resulted in a significant reduction of VEGF-A_165_-induced angiogenesis when compared to cells transfected with a miR negative control (Fig. S1B).

These data demonstrate that the two strands of miRNA-133a inhibit endothelial cell angiogenesis to different degrees, suggesting that they target different angiogenic cellular pathways. These results indicate a prominent role for miR-133a-3p as a negative regulator of endothelial cell angiogenesis.

### Aberrant expression of miR-133a-3p alters VEGF-A_165_-induced Notch signalling in endothelial cells

**Figure 2:**
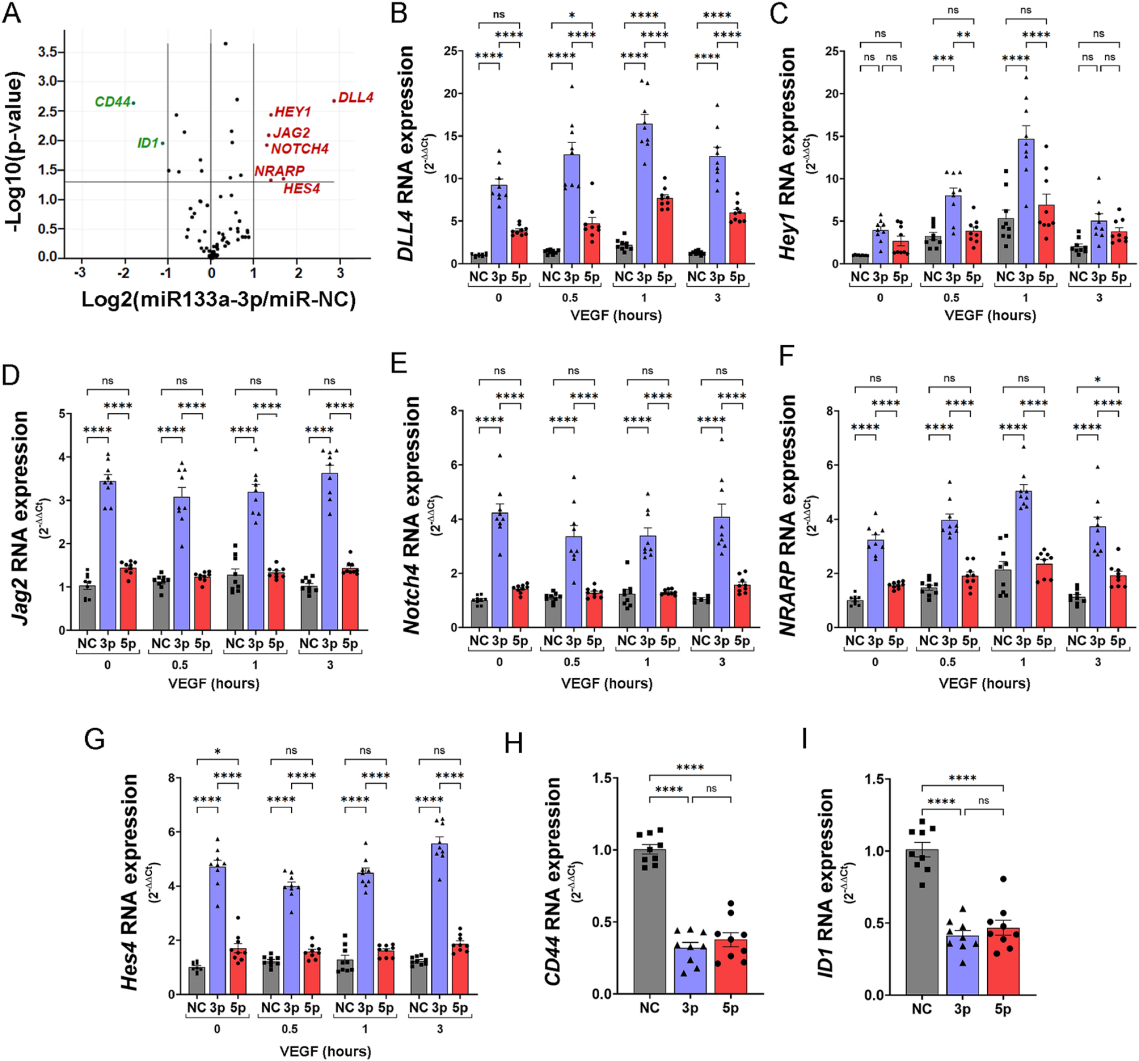
Aberrant expression of miR-133a in endothelial cells alters the expression of key regulators of DLL4-Notch signalling. (**A**) Volcano plot representation showing differential gene expression analysis of a RT2 Profiler PCR Array Human Notch Signalling Pathway Plus Kit (Ref PAHS-059Y, Qiagen) using RNA isolated from HUVEC transfected with miR-133a-3p or negative control (miR-NC) and stimulated for 1 h with VEGF-A_165_ (50 ng/ml). Results of three independent experiments were analysed using GeneGlobe Data Analysis Center (Qiagen). Genes showing a Log2(miR-133a-3p/miR-NC) > 1 and − Log10(*P* value) *P* ≤ 0.05 were selected as upregulated (red). Those with Log2(miR-133a-3p/miR-NC) < -1, and − Log10(*P* value) *P* ≤ 0.05 were selected as downregulated (green). (**B**–**I**) HUVEC transfected with mimics for negative control (NC, grey bars), miR-133a-3p (3p, blue bars) or miR-133a-5p (5p, red bars) were left unstimulated (0 h) or stimulated with VEGF-A_165_ (50 ng/ml) for the indicated times. RNA isolated from these cells were used to determine changes in gene expression using qPCR TaqMan Gene Expression Assays specific for each gene. Data are shown as mean ± SE, n = 9. Data were analysed for statistical differences by two-way (**B**–**G**) or one-way (**H**–**I**) ANOVA with post hoc Tukey’s comparison test. ns = non-significant; *, *P* ≤ 0.05; **, *P* ≤ 0.01; ***, *P* ≤ 0.001; ****, *P* ≤ 0.0001.

A large body of data has shown that members of the Notch signalling pathway play a pivotal role as regulators of sprouting angiogenesis induced by VEGF^18^. Given the strong inhibitory effect observed for miR-133a-3p in endothelial cell tubular morphogenesis (Fig. 1, Fig. S1), we decided to transfect HUVEC with miR-133a-3p mimics (or miR-NC as a control) and analyse the expression of genes encoding members of the Notch signalling pathway after VEGF-A_165_ stimulation. PCR-based screening of an array containing primers to detect the expression of a selection of Notch-related genes showed that ectopic expression of miR-133a-3p in VEGF-A_165_-stimulated cells promoted a significant increase in the expression of *DLL4*, *Hey1*, *Jag2*, *Notch4*, *NRARP,* and *Hes4* (Fig. 2A and Supplementary Table S1). To validate these results, and to further investigate whether the strand miR-133a-5p also had any effect on the regulation of these genes, we carried out a new set of transfections in HUVEC stimulated with VEGF-A_165_ at several time points. TaqMan-based qPCR analysis confirmed the data obtained in the gene array screening. Ectopic expression of miR-133a-3p significantly increased the expression of these genes in both basal and VEGF-A_165_-stimulated conditions (Fig. 2B–G). In contrast, miR-133a-5p overexpression did not have any major effect on the expression of these genes, except for *DLL4* which was increased in miR-133a-5p transfected cells but only after stimulation with VEGF-A_165_ (Fig. 2B–G).

To confirm that the effect of miR-133a-3p on the expression of Notch-signalling genes observed in HUVEC is extended to endothelial cells isolated from a different vascular bed, we transfected primary Human Aortic Endothelial Cells (HAoEC) with miR-133a-3p or miR-NC (negative control) and isolate RNA from the transfected cells. Subsequent qPCR experiments revealed that miR-133a-3p significantly upregulated the expression of *DLL4*, *Jag2* and *Notch4* in a similar way to that found in HUVEC cells (Fig. S2A–C).

It is well established that activation of DLL4/Notch signalling negatively modulates sprouting angiogenesis^18,19^. Thus, our data suggest that enhanced expression of members of this pathway is one of the factors mediating the inhibitory effect exerted by miR-133a-3p in angiogenesis.

Opposite to the increase in gene expression described above, gene array screening also revealed that miR-133a-3p led to a significant decrease in the expression of *CD44* and *ID1* (Fig. 2A and Supplementary Table S1). Further qPCR TaqMan-based analyses were conducted and validated these observations. In this case, aberrant expression of either, miR-133a-3p or -5p, significantly inhibited the expression of both genes to a similar extent (Fig. 2H–I). CD44 and ID1 have been described as strong stimulators of endothelial angiogenesis^20,21^. Thus, decreased levels of *CD44* and *ID1* expression in miR-133a expressing cells are concurrent with the negative effect of miR-133a on endothelial cell tubular morphogenesis.

### Ectopic expression of miR-133a-3p or -5p decreases endothelial cell proliferation

**Figure 3:**
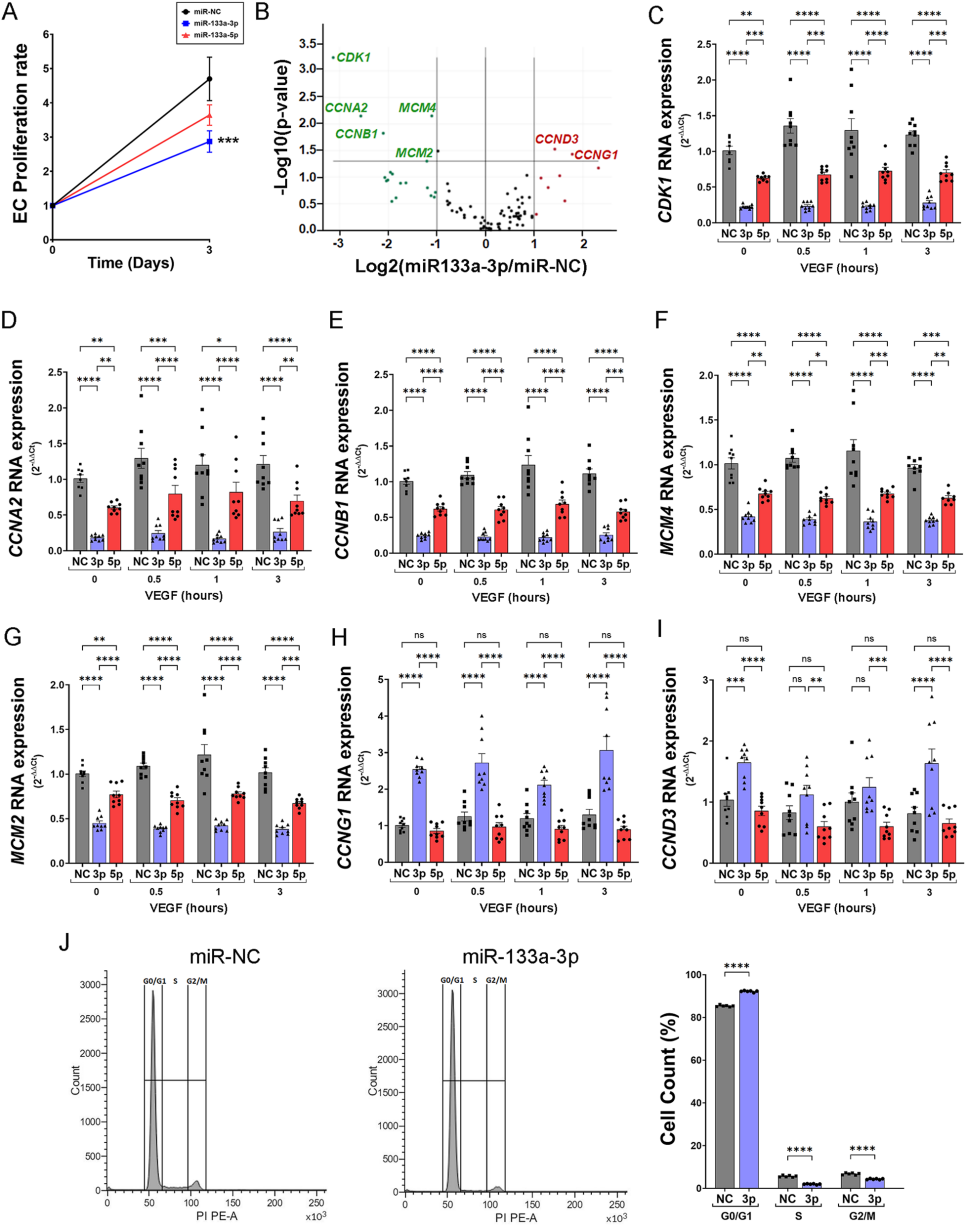
miR-133a strands -3p and -5p attenuate endothelial cell proliferation. HUVEC were transfected with mimics for miR negative control (miR-NC) or mimics for miR-133a strands -3p (miR-133a-3p) or -5p (mir-133a-5p) and were left unstimulated (0 h) or stimulated with VEGF-A_165_ as indicated. (**A**) Proliferation rate of transfected cells assayed by MTT. Proliferation rate was calculated as absorbance in MTT assay after 3 days divided by absorbance at 0 days. Data are shown as mean ± SE, n = 8. ***, *P* ≤ 0.001 miR-133a-3p versus miR-NC at 3 Days analysed by two-way ANOVA with post hoc Tukey’s comparison test. (**B**) Volcano plot representation showing differential gene expression analysis of a RT2 Profiler PCR Array Human Cell Cycle Kit (Ref PAHS-020Z, Qiagen) using RNA isolated from HUVEC transfected with miR-133a-3p or negative control (miR-NC) and stimulated for 1 h with VEGF-A_165_ (50 ng/ml). Results of three independent experiments were analysed using GeneGlobe Data Analysis Center (Qiagen). Genes showing a Log2(miR-133a-3p/miR-NC) > 1 and − Log10(*P* value) *P* ≤ 0.05 were selected as upregulated (red). Those with Log2(miR-133a-3p/miR-NC) < − 1, and − Log10(*P* value) *P* ≤ 0.05 were selected as downregulated (green). (**C**–**I**) HUVEC transfected with mimics for negative control (NC, grey bars), miR-133a-3p (3p, blue bars) or miR-133a-5p (5p, red bars) were left unstimulated (0 h) or stimulated with VEGF (50 ng/ml) for the indicated times. RNA isolated from these cells were used to determine changes in gene expression using qPCR TaqMan Gene Expression Assays specific for each gene. Data are shown as mean ± SE, n = 9. Data were analysed for statistical differences by two-way ANOVA with post hoc Tukey’s comparison test. ns = non-significant; *, *P* ≤ 0.05; **, *P* ≤ 0.01; ***, *P* ≤ 0.001; ****, *P* ≤ 0.0001. (**J**) Flow cytometry cell cycle analysis of HUVEC transfected with negative control (miR-NC) or miR-133a-3p mimics. The proportion of HUVECs in each phase of the cell cycle was determined by quantification of DNA content using propidium iodide. Representative cell count/PI plots are shown. Data are shown as mean ± SE, n = 6. ****, *P* ≤ 0.0001 two-way ANOVA with post hoc Tukey’s comparison test for the indicated groups.

The proliferation of endothelial cells in response to pro-angiogenic stimuli is a pivotal process during the formation of new blood vessels. To investigate whether miR-133a negatively affects endothelial cell proliferation, we transfected HUVEC with miR-133a-3p or -5p mimics and compared their proliferation to that in cells transfected with control mimic miR-NC. As shown in Fig. 3A, both miR-133a-3p and miR-133a-5p diminished endothelial cell proliferation, although a more pronounced inhibition was produced by the -3p strand (Fig. 3A). Although the MTT assay used in these experiments is often used as a surrogate measure of cell proliferation, this assay determines the enzymatic activity of mitochondrial enzymes in healthy cells. To confirm the negative effect of miR-133a on endothelial cell proliferation, we infected HUVEC with an adenovirus expressing both strands of miR-133a (3p and 5p) (AdmiR-133a) or a control adenovirus that does not express any miR (AdmiR-NC) and quantify the number of cells after 3 days of culture. In concurrence with our results using MTT assay, ectopic expression of miR-133a strongly inhibited HUVEC proliferation (Fig. S3A). To further reinforce this result, we repeated the experiment in endothelial cells isolated from an alternative vascular bed. Ectopic expression of miR-133a in HAoEC also resulted in a significant decrease in the number of cells after 3 days of culture (Fig. S3B).

To gain insights into the molecular mechanisms responsible for this decrease in cell proliferation, we isolated RNA from HUVEC cells transfected with miR-133a-3p or miR-NC and stimulated with VEGF-A_165_ for 1 h, then used it to screen a gene array containing primers to detect the expression of a selection of cell cycle-related genes (Qiagen). Ectopic expression of miR-133a-3p led to strong downregulation in the expression of genes encoding cell cycle regulators such as cyclin-dependent kinase 1 (*CDK1*), cyclin A2 (*CCNA2*) and cyclin B1 (*CCNB1*), and initiators of genome replication such as mini-chromosome maintenance protein 2 (*MCM2*) and 4 (*MCM4*) (Fig. 3B and Supplementary Table S2). Genes encoding cyclin D3 (*CCND3*) and G1 (*CCNG1*) were however upregulated by the presence of miR-133a-3p (Fig. 3B and Supplementary Table S2). To further investigate the role of each miR133a strand in the expression of these genes, we conducted independent transfections with either the -3p or the -5 strands and stimulated the transfected cells with VEGF-A_165_ for several periods. qPCR analysis of RNA isolated from these cells confirmed the results obtained by gene array, and further indicated that ectopic expression of miR-133a-3p led to a reduction in the expression of *CDK1*, *CCNA2*, *CCNB1*, *MCM4*, *MCM2* and an increase in *CCNG1* and *CCND3* (Fig. 3C–I). In all cases, miR-133a-3p related changes in gene expression were independent of VEGF-A_165_ treatment and were observed in unstimulated cells, although they were maintained after stimulation. Equivalent results were obtained when we assayed the effect of miR-133a-3p ectopic expression in HAoEC. Ectopic expression of miR-133a-3p resulted in a significant reduction of *CDK1*, *CCNA2*, *CCNB1*, *MCM2* RNA levels in an VEGF-A_165_-independent manner (Fig. S2F–I).

Transfection of miR-133a-5p mimic also had a downregulatory effect on the genes inhibited by -3p (Fig. 3C–G) but did not change the expression of *CCNG1* and *CCND3*, suggesting that these two genes are specifically targeted by the -3p strand (Fig. 3H–I).

Cyclins and MCM proteins play a central role in regulating entry into the S phase and progression of DNA replication during the cell cycle^22^. Flow cytometer analysis in the population of HUVEC transfected with miR-133a-3p showed a higher percentage of cells in G0/G1, but less in the S and G2/M phases of the cell cycle, when compared to control cells transfected with miR-NC (Fig. 3J). These results are likely to be the consequence of the miR-133a-3p mediated changes in the expression of key cell cycle regulators described above and are concurrent with the observed decrease in cell proliferation.

### Ectopic expression of miR-133a-3p reduces endothelial cell motility

**Figure 4:**
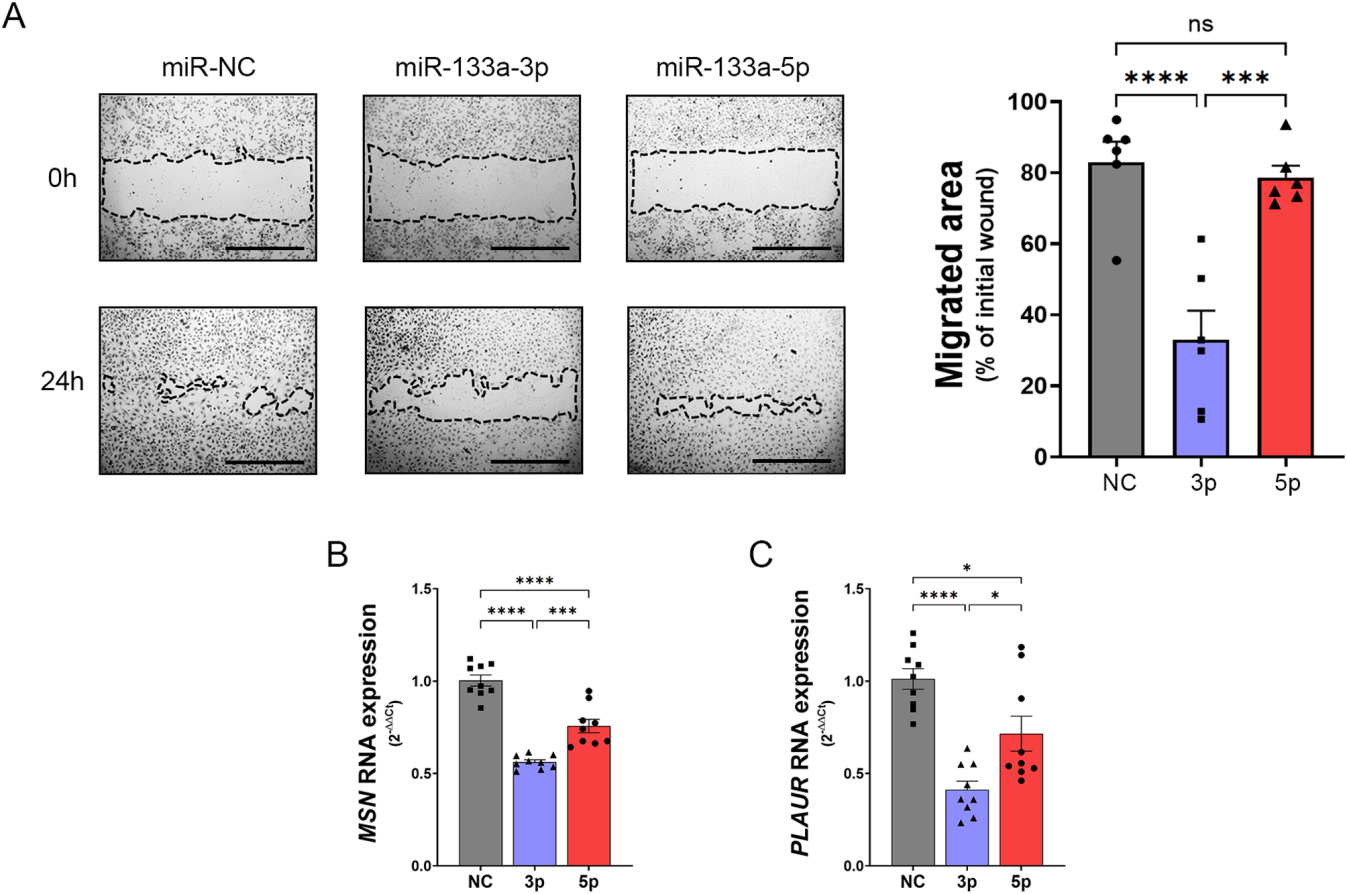
Aberrant expression of miR-133a-3p strongly inhibits endothelial cell migration. (**A**) Representative images of wound-healing migration assays performed with HUVEC transfected with microRNA mimics for miR-133a-3p, miR-133a-5p or a negative control (miR-NC). Images were taken at time zero (0 h) and after incubation for 24 h (24 h). The migrated area was calculated using ImageJ software (https://imagej.nih.gov/ij/) by subtracting the value of the non-migrated area from the wound area at time zero and expressing this as a percentage of the total area at time zero. Scale bars, 1000 μm. Data are shown as mean ± SE, n = 6. Data were analysed for statistical differences by one-way ANOVA with post hoc Tukey’s comparison test. ns = non-significant; ***, *P* ≤ 0.001; ****, *P* ≤ 0.0001. (**B**,**C**) TaqMan gene expression assay analysis of RNA levels for *MSN* and *PLAUR* in HUVEC transfected with microRNA mimics as above. Data are shown as mean ± SE, n = 9. *, *P* ≤ 0.05; ***, *P* ≤ 0.001; ****, *P* ≤ 0.0001 one-way ANOVA with post hoc Tukey’s comparison test for the indicated groups.

The migration of endothelial cells towards angiogenic cues such as VEGF-A_165_ is a well-established requisite for the sprouting of new blood vessels^23^. Thus, we decided to analyse the effect of miR-133a-3p and -5p overexpression on endothelial cell motility by performing wound-healing migration assays with HUVEC transfected with mimics of these miRs. The transfection of miR-133a-3p into HUVEC strongly attenuated the motility of the cells compared to that in control cells transfected with miR-NC (Fig. 4A). However, the expression of miR-133a-5p did not induce any significant changes in the motility of the cells (Fig. 4A). To confirm that the differences observed in these experiments were truly the consequence of cell migration inhibition, we repeated the experiments in HUVEC and HAoEC but in this case the cells were incubated for 24 h in the presence of 2 mM hydroxyurea to inhibit cell proliferation (Fig. S4). In these conditions, ectopic expression of miR-133a again resulted in diminished migration of HUVEC (Fig. S4A) and HAoEC (Fig. S4B).

Some previously identified miR-133a target genes, such as *moesin*, encode proteins that play a pivotal role in endothelial cell migration and tubular morphogenesis^24,25^. Notably, endothelial moesin has been identified as an essential mediator of uPAR-dependent chemotaxis and tube formation in endothelial cells^26^. This information prompted us to investigate whether miR-133a induced any changes in *moesin* and *uPAR* expression in the transfected cells. Taqman qPCR assays showed that the RNA levels of *moesin* and *uPAR* notably decreased in HUVEC transfected with miR-133a-3p (Fig. 4B,C). miR-133a-5p also reduced *moesin* and *uPAR* expression but not to the extent observed with -3p (Fig. 4B,C). Additional experiments performed in HAoEC also showed reduced mRNA levels for *moesin* and *uPAR* in cells transfected with miR-133a-3p, indicating that the downregulation of the expression of these genes mediated by miR-133a-3p happens in endothelial cells isolated from several vascular beds (Fig. S2D,E).

These results suggest that the aberrant expression of miR-133a-3p suppresses the expression of key mediators of endothelial cell migration such as the axis uPAR-Moesin.

### miR-133a-3p regulates the expression of endothelial genes related to extracellular matrix remodelling

**Figure 5:**
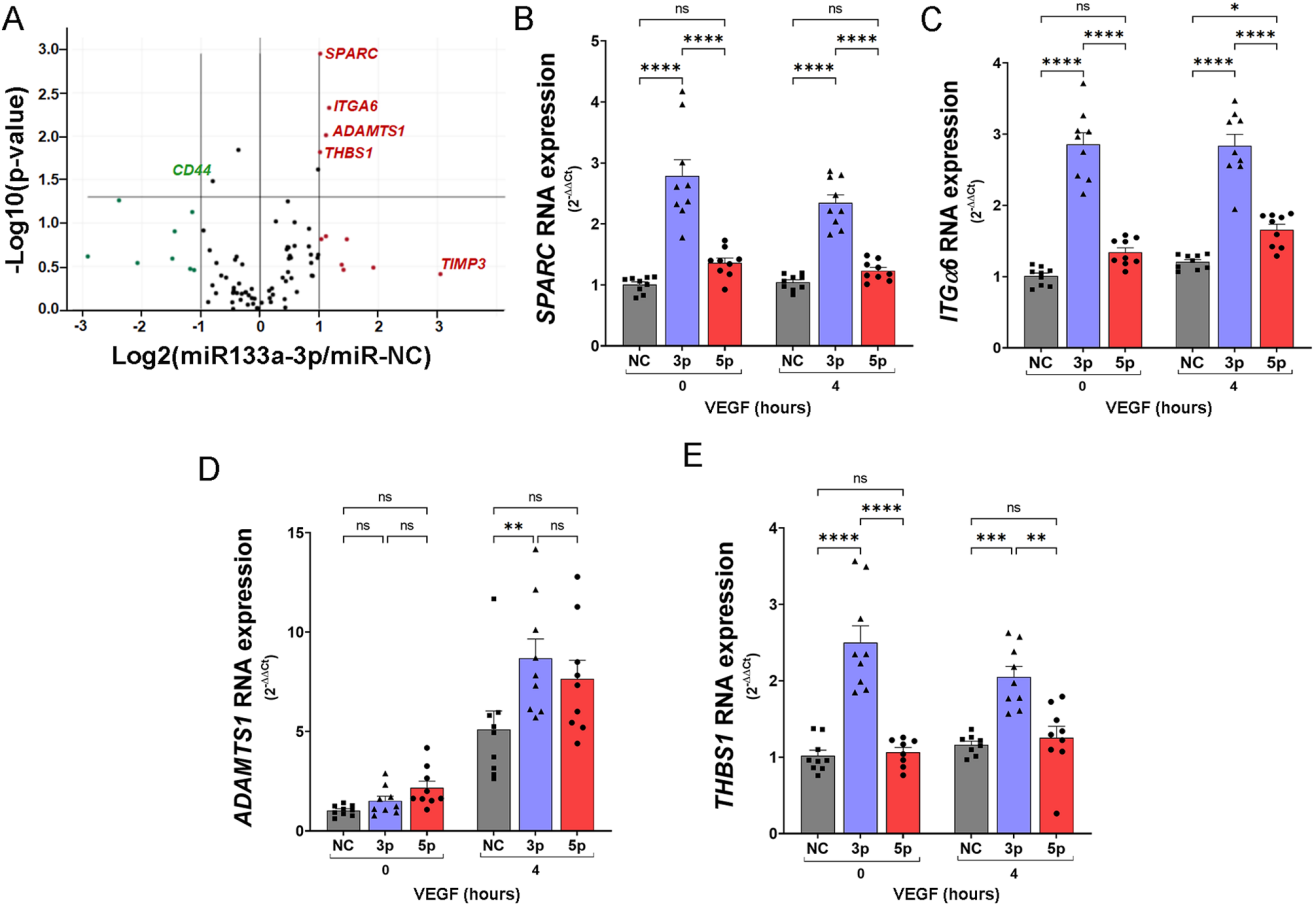
Ectopic expression of miR-133a-3p alters the expression of key mediators of ECM remodelling. (**A**) Volcano plot representation showing differential gene expression analysis of a RT2 Profiler PCR Array Human Extracellular Matrix & Cell Adhesion Molecules Kit (Ref PAHS-013Z, Qiagen) using RNA isolated from HUVEC transfected with miR-133a-3p or negative control (miR-NC) and stimulated for 4 h with VEGF-A_165_ (50 ng/ml). Results of three independent experiments were analysed using GeneGlobe Data Analysis Center (Qiagen). Genes showing a Log2(miR-133a-3p/miR-NC) > 1 and − Log10(*P* value) *P* ≤ 0.05 were selected as upregulated (red). Those with Log2(miR-133a-3p/miR-NC) < − 1, and − Log10(*P* value) *P* ≤ 0.05 were selected as downregulated (green). (**B**–**E**) HUVEC transfected with mimics for negative control (NC, grey bars), miR-133a-3p (3p, blue bars) or miR-133a-5p (5p, red bars) were left unstimulated (0 h) or stimulated with VEGF-A_165_ (50 ng/ml) for 4 h. RNA isolated from these cells were used to determine changes in gene expression using qPCR TaqMan Gene Expression Assays specific for each gene. Data are shown as mean ± SE, n = 8. Data were analysed for statistical differences by two-way ANOVA with post hoc Tukey’s comparison test. ns = non-significant; *, *P* ≤ 0.05; **, *P* ≤ 0.01; ***, *P* ≤ 0.001; ****, *P* ≤ 0.0001.

Endothelial cell migration and the formation of new vessel sprouts towards pro-angiogenic signals is not possible without previous remodelling of the extracellular matrix (ECM) induced by secreted proteases in the area surrounding vessel branching^27^. To investigate whether the presence of miR-133a in endothelial cells alters the expression of components of the ECM and degrading proteases, we transfected HUVEC with mimics miR-NC or miR-133a-3p. The transfected cells were treated with VEGF-A_165_ (50 ng/ml) for 4 h, and the RNA isolated from these cells was used to screen a gene array containing primers to detect the expression of genes encoding for ECM proteins or related proteases (Qiagen). Transfection of miR-133a-3p enhanced the expression of *SPARC* (Secreted Protein Acidic and Cysteine Rich), *ITGA6* (Integrin subunit alpha 6), *ADAMTS1* (ADAM Metallopeptidase with Thrombospondin Type 1 Motif 1), and *THBS1* (Thrombospondin 1) (Fig. 5A and Supplementary Table S3). To validate these data and extend our initial analyses, we repeated the transfection experiments including a new group of cells transfected with miR-133a-5p. Moreover, in all groups, transfected cells were left unstimulated (0 h) or treated with VEGF-A_165_ for 4 h (4 h). Gene expression was determined by qPCR using TaqMan gene expression assays specific for each gene. As shown in Fig. 5B–E, miR-133a-3p enhanced the expression of *SPARC*, *ITGA6*, and *THBS1* in unstimulated and VEGF-A_165_-stimulated cells to the same degree, indicating that the effect was not dependent on stimulation. *ADAMTS1* gene expression was also augmented by transfection of miR-133a-3p but only after stimulation with VEGF-A_165_ (Fig. 5D). The expression of these genes was not affected by the ectopic expression of miR-133a-5p (Fig. 5B–E). Further experiments performed in endothelial cells isolated from a different vascular bed, HAoEC, corroborated enhanced expression of *SPARC* and *THBS1* in the presence of ectopic miR-133a-3p (Fig. S2J,K).

### Bioinformatic analysis of differential gene expression induced by miR-133a-3p in HUVEC predicts a decrease in pro-angiogenic cellular functions

The analyses of gene arrays for Notch-signalling regulators, cell cycle, or ECM-CAM that we have described above revealed differential expression of 23 genes in endothelial cells aberrantly expressing miR-133a-3p (Supplementary Table S4). Additionally, we decided to investigate the effect of miR-133a-3p on the expression of *TIMP3* using TaqMan gene assay, as the ECM-CAM gene array analysis showed very strong upregulation of this gene although with a low *P* value (Fig. 5A and Supplementary Table S3). TaqMan gene expression assays for *TIMP3* in HUVEC transfected with miR-133a-3p showed strong upregulation of this gene in basal and VEGF-A_165_-stimulated conditions when compared to its expression in control cells transfected with miR-NC (Fig. S5A). Additionally, we also examined the expression of *RCAN1.4*, a gene induced by VEGF-A_165_ stimulation in endothelial cells with a well-established role as a negative regulator of angiogenesis^28^. Ectopic expression of miR-133a-3p strongly enhanced VEGF-A_165_-mediated upregulation of *RCAN1.4* (Fig. S5C) suggesting this gene is an important mediator of the inhibitory effect exerted by miR-133a-3p on angiogenesis. Additional experiments performed in HAoEC showed equivalent results when the expression of these genes was analysed in cells expressing miR-133a-3p (Fig. S5B,D).

**Figure 6:**
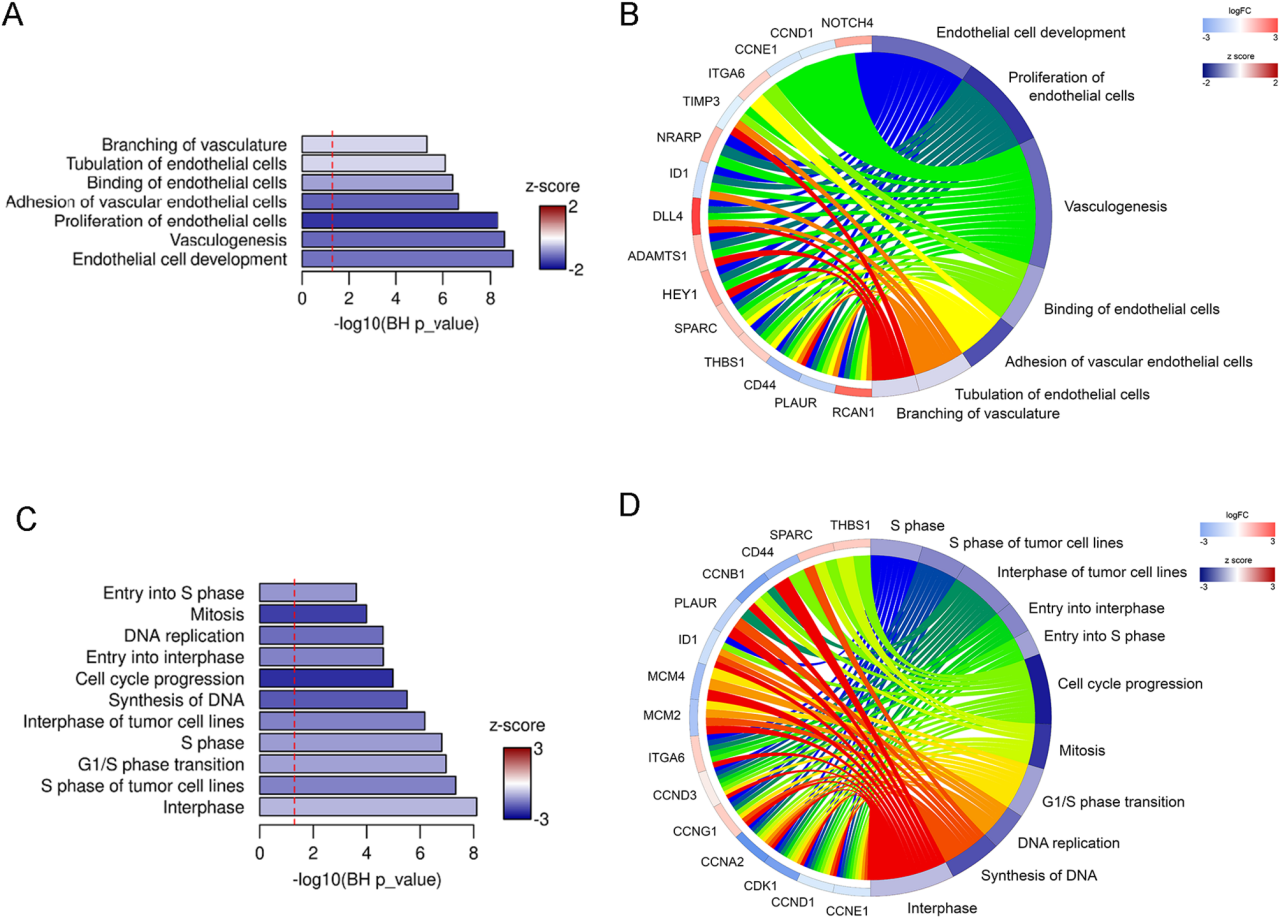
Ingenuity Pathway Analysis of differentially expressed genes in endothelial cells expressing ectopic miR-133a-3p. (**A**,**C**) Bar plots showing enriched biological functions predicted by IPA in the category “Cardiovascular system development and function” (**A**) and “Cell Cycle”, and “DNA replication, recombination and repair” (**C**). Bar lengths are relative to enrichment significance, expressed as − log10(Benjamini–Hochberg adjusted *P* values). (**B**,**D**) Circular plots representing enriched biological functions detected in the categories “Cardiovascular system development and function” (**B**) and “Cell Cycle”, “DNA replication, recombination and repair” (**D**). Enriched biological functions are connected to the associated differentially expressed genes. FC, fold change. Activation z-score scale: blue repression; red, activation. LogFC scale: red, upregulated; blue, downregulated. Barplots were generated with R 3.6.3 (https://www.r-project.org). Circular plots were generated with GOplot 1.0.2 (https://wencke.github.io).

We then decided to investigate whether the differential gene expression profile described for this set of 25 genes would underline the inhibitory effect in angiogenesis mediated by miR-133a-3p (Supplementary Table S4). IPA analysis highlighted enrichment of cellular functions included in the categories “Cardiovascular system development and function”, “Cell Cycle”, and “DNA replication, recombination and repair” (Fig. 6). Interestingly, activation Z-scored predicted decreased cellular functions such as “Branching of vasculature”, “Tubulation of endothelial cells”, “Vasculogenesis”, “Proliferation of endothelial cells”, “Cell cycle progression”, “Mitosis”, “DNA replication” and “Synthesis of DNA” (Fig. 6) that support our results on the inhibitory role of miR-133a-3p in angiogenic cellular processes.

### Ectopic expression of miR-133a-3p decreases pro-angiogenic but increases anti-angiogenic proteins in endothelial cells

**Figure 7:**
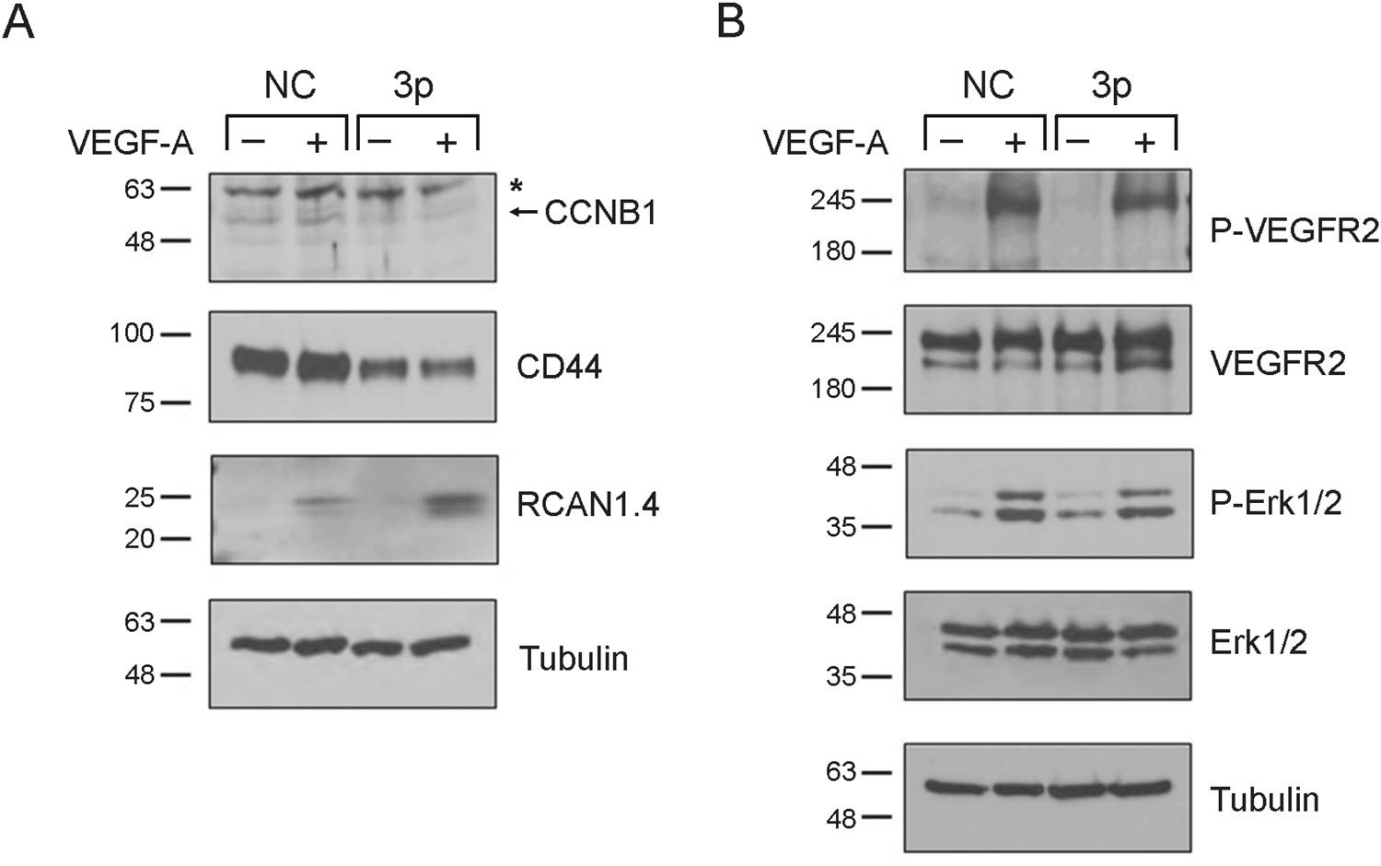
Ectopic expression of miR-133a-3p results in a decrease of pro-angiogenic and an increase in anti-angiogenic proteins in endothelial cells. (**A**) HUVEC were transfected with a control miR mimic (NC) or a mimic for human miR-133a-3p (3p) and were left unstimulated (−) or were stimulated with VEGF-A_165_ (50 ng/ml) for 4 h (+). Expression of CCNB1, CD44 and RCAN1.4 was determined by western blot analyses of total proteins isolated from the transfected cells. Levels of Tubulin were determined in the same samples for loading control. Blot images are representative of two independent experiments. The band corresponding to Cyclin B1 is pointed out by an arrow. *, denotes unspecific binding. (**B**) HUVEC were transfected as in (**A**) and then stimulated (+) or not (−) with VEGF-A_165_ (50 ng/ml) for 5 min. The expression and phosphorylation/activation status of VEGFR2 and Erk1/2 proteins was determined by western blot analysis of total proteins isolated from the transfected cells. Blot images are representative of two independent experiments. Levels of Tubulin were determined in the same samples for loading control.

Our results clearly indicate that aberrant expression of miR-133a-3p increases anti-angiogenic but decreases pro-angiogenic gene expression in endothelial cells. To shed further insights into the intracellular mechanisms implicated in miR-133a-3p- dependent inhibition of angiogenesis, we transfected HUVEC with miR-NC or miR-133a-3p mimics and analysed by western blot the effect of ectopic miR-133a-3p on the levels of angiogenic regulators at the protein level. Reinforcing the relevance of the changes in gene expression identified in this study, changes in the expression of RNAs for angiogenic activators (decrease of *CCNB1* or *CD44*), or angiogenic inhibitors (increase of *RCAN1.4*) induced by ectopic expression of miR-133a-3p, correlate with changes at the protein level (Fig. 7A).

To identify other potential mechanisms implicated on the effect of miR-133a on angiogenesis, we have analysed its effect on the expression and functionality of members of the VEGF-A/VEGFR2 axis. Our results demonstrate that ectopic expression of miR-133a-3p do not affect the expression levels of VEGFR2 or its potential phosphorylation/activation by VEGF-A_165_ (Fig. 7B). Likewise, activation/phosphorylation of the signalling protein Erk1/2 in response to VEGF-A_165_ stimulation was identical in the presence or absence of miR-133a-3p (Fig. 7B). These results agree with a bioinformatic analysis performed using the databases miRWalk, Target-Scan, and miRDB to identify potential miR-133a binding sites in the genes encoding components of the VEGF/VEGFR signalling axis. No potential binding sites for miR-133a were predicted simultaneously by the three databases when the sequences of *VEGF-A*, *VEGF-B*, *VEGF-C*, *VEGFR1*, *VEGFR2*, or *VEGFR3* were analysed (Supplementary Table S5). Further analysis on the sequences of genes encoding signalling effectors of this axis such as *Erk*, *JNK*, *p38*, *Calcineurin-A*, *Calcineurin-B*, *NFAT1*, *NFAT2*, *NFAT3* or *NFAT4*^29–31^ did not reveal any potential miR-133a targeting simultaneously identified by the three databases (Supplementary Table S5). The only gene where potential binding sites for miR-133a were predicted by the three data bases was *NFAT5* (Supplementary Table S5). However, NFAT5 has not been described as an effector of VEGF signalling in endothelial cells. NFAT5 has been described as a mediator of arteriogenesis and angiogenesis via MCP1-dependent monocyte recruitment to ischaemic limbs^32^ but its role in VEGF-A_165_-induced angiogenesis is not fully understood at present.

These results suggest that the inhibitory effect of miR-133a on VEGF-induced angiogenesis is not exerted by altering the expression or functionality of the VEGF-/VEGFR axis, and involves changes in the expression of genes activated by VEGF-A_165_ stimulation of VEGFR2. These results reinforce the functional relevance of the miR-133a-mediated changes in the expression of the genes identified on this study.

## Discussion

Here we show that the expression of miR-133a inhibits endothelial cell proliferation, migration, and tubular morphogenesis, with a stronger effect of miR-133a-3p over the -5p strand. Moreover, we describe a collection of genes differentially expressed in endothelial cells transfected with miR-133a-3p that underpin its negative effect on angiogenesis.

In agreement with the inhibitory role on angiogenesis, aberrant expression of miR-133a-3p in primary HUVEC strongly enhanced the expression of several components of the Notch signalling pathway such as *DLL4* (16.4-fold increase), *Hey1* (14.7-fold increase), and *Notch4* (3.15-fold increase) (Fig. 2) with a well-established anti-angiogenic function^33–36^. Similarly, miR-133a-3p led to a significant increase in the expression of genes related to ECM remodelling such as *SPARC* (2.79-fold increase), *THBS1* (2.5-fold increase), *ADAMTS1* (8.68-fold increase) (Fig. 5) and *TIMP3* (5.72-fold increase) (Fig. S5A) that have also been described as robust angiogenic inhibitors^37–40^. Moreover, we show that the expression of *RCAN1.4*, another angiogenesis inhibitor, is massively upregulated (67.7-fold increase) in VEGF-A_165_-stimulated HUVEC transfected with miR-133a-3p (Fig. S5C). In parallel to the enhancement of anti-angiogenic genes, miR-133a-3p led to a decrease in the expression of pro-angiogenic genes such as *CD44* (68% reduction), *ID1* (59% reduction), *CDK1* (78% reduction), *CCNA2* (83% reduction), *CCNB1* (77% reduction), *MCM2* (63% reduction), *MCM4* (64% reduction), *MSN* (45% reduction) and *PLAUR* (58% reduction)^21,41–43^. Furthermore, Ingenuity Pathway Analysis of the changes in endothelial gene expression detected in this study predicts that ectopic expression of miR-133a-3p in endothelial cells leads to a decrease in essential pro-angiogenic cellular functions (Fig. 6).

Equivalent results were observed when we repeated these experiments in HAoEC (Fig. S2, S5) indicating that the observed changes in gene expression described in this work occur in endothelial cells isolated from different vascular beds.

Western blot experiments have shown that decrease in RNA levels for angiogenic activators *CNNB1* or *CD44* mediated by miR-133a-3p correlate with changes at the protein level (Fig. 7). Likewise, enhancement in the expression of the anti-angiogenic gene *RCAN1.4* is also translated to increased expression of RCAN1.4 protein in endothelial cells expressing miR-133a-3p (Fig. 7).

Altogether, these results support the negative effect on angiogenesis exerted by miR-133a-3p described in this work.

Our data are concurrent with the observations by Chen et al. (2018) and Soufi-Zomorrod et al. (2016)^11,12^. However, they contradict the results by Zhu et al. (2021) that describe a positive regulatory role for miR-133a-3p in angiogenesis^13^. Our experiments show the effect of a direct transfection of miR-133a-3p into endothelial cells. We have followed this approach as the aberrant expression of miR-133a in endothelial cells was originally described by Li et al. (2016) as the consequence of transcriptional activation of the *miR-133a* promoter^10^, therefore we aimed to mimic the conditions occurring in the diseased endothelium. In their work, Zhu et al. use mesenchymal stem cells infected with a lentivirus overexpressing the macrophage migration inhibitory factor protein to produce exosomes rich in miR-133a-3p, and thus, other components of the exosomal cargo might be causing the discrepancy about the effect exerted by miR-133a-3p on angiogenesis.

In silico analyses has predicted potential binding sites for miR-133a-3p in the 3’UTR of *MSN*^24^ that might explain the observed decrease in RNA levels. The molecular mechanisms exerted by miR-133a-3p to alter the expression of the other genes described in this work is unknown at this point and beyond the scope of this paper. It is tempting to speculate that the decrease in gene expression might be the result of the direct binding of miR-133a-3p to complementary motifs in the sequence of the targeted gene. However, bioinformatics analysis using the databases miRWalk, Target-Scan, and miRDB showed that apart from *MSN*, where potential binding sites were predicted by TargetScan and miRDB (but not miRWalk), none of the other genes showed any potential binding sites predicted by at least two data bases (Supplementary Table S6). Nevertheless, miR-133a-3p may downregulate gene expression by recognising sequences that are not predicted by the data bases. In addition to the canonical post-transcriptional repression exerted by miRs binding to target RNAs in the cytoplasm, miRs have also been reported to regulate transcriptional gene expression in the nucleus by binding to specific sequences in the promoter of target genes^44^. In fact, nuclear re-localisation of miR-133a has been found to repress the transcriptional expression of the de novo* DNA methyltransferase 3B* (*Dnmt3b*) by recognising a complementary sequence in its promoter^45^. Thus, miR-133a might be operating a similar mechanism to downregulate expression of the genes described here.

We also show that miR-133a enhances the expression of inhibitors of angiogenesis. Mechanistically, these results could be attributed to an indirect effect provoked by miR133a-mediated inhibition of a repressor of the augmented RNA. This mechanism has been described for example for some miR-1 effects. In zebrafish embryos, miR-1 inhibits the expression of SARS, a transcriptional repressor of the *VEGFaa* gene, leading to increased VEGFaa expression^46^. Another possibility is the direct action of nuclear miRs that recognise complementary regions in the promoter of target genes. This has also been reported as a mechanism of action of some miRs that enhance transcriptional gene expression^47^. The molecular mechanisms employed by miR-133a to alter the expression of the genes described in this study require further investigation.

In conclusion, our results describe that the aberrant expression of miR-133a in endothelial cells inhibits blood vessel formation by altering the expression of a network of target genes encoding key regulators of angiogenesis. Given the relevance of angiogenesis in the progression of human diseases occurring with excessive angiogenesis, the specific delivery of miR-133a-3p mimics to endothelial cells within tumours or the neo-vasculature of diseased diabetic eyes, might open new therapeutic interventions to treat these patients. Although the potential therapeutic delivery of miR-133a-3p requires validation using animal models of pathological angiogenesis, our results warrant further investigations into this possibility.

## Methods

### Cells and cell culture

Human umbilical vein endothelial cells (HUVEC) were purchased from TCS Cellworks or PromoCell and cultured in tissue culture flasks pre-coated with 0.1% gelatin in endothelial cell growth medium (ECGM, PromoCell) supplemented with ECGM-supplement mix and 1% penicillin/streptomycin/amphotericin B (Sigma-Aldrich). HUVEC were used at passages 6–8.

Human cardiac microvascular endothelial cells (HCMEC) were purchased from PromoCell and cultured as described above. HCMEC were used at passages 4–6.

Human Aortic Endothelial Cells (HAoEC) were purchased from Promocell and cultured as described above. HAoEC were used at passages 6–8.

### miRNA mimic transfection in endothelial cells

miRNA “mimic” for human miRNA-133a-3p (NBS Biologicals, ref: MCH01280), human miRNA-133a-5p (NBS Biologicals, ref: MCH01281), or a miRNA “mimic” negative control miRNA-NC (NBS Biologicals, ref: MCH00000) were transfected into primary HUVEC as following. To achieve maximal stimulation with VEGF-A_165_, HUVEC were cultured in tissue culture plates without gelatin coating for 2 passages before transfection. For transfection, HUVEC were plated in 6-well tissue culture plates without gelatin pre-coating (3 × 10^5^ cells/well) and incubated overnight. The following morning, cells were washed twice with PBS and incubated in serum-free, antibiotic-free OPTIMEM medium for 1 h. Then, 100 pmol of miRNA “mimic” were incubated for 20 min with 5 μl of Lipofectamine 2000 (Invitrogen) in 500 μl of OPTIMEM and added to the cells. Transfection medium was removed after incubation for 6 h and substituted by ECGM supplemented with ECGM-supplement mix and 1% penicillin/streptomycin/amphotericin B (Sigma-Aldrich). Cells were incubated for 72 h and then used for further experiments.

HAoEC were transfected as indicated for HUVEC.

For stimulation, cells were washed in PBS and starved in serum-free ECGM for 3 h before treatment with VEGF-A_165_ (50 ng/ml) (Peprotech) for the indicated time.

### Adenoviral-mediated expression of miR-133a

HUVEC or HAoEC plated in 6-well tissue culture plates (3 × 10^5^ cells/well) were incubated overnight to attach. The following morning, cells were infected with an adenovirus expressing miR-133a (Ad-miR133a) or a control adenovirus expressing no miR (Ad-miRNC) at an MOI = 200 and incubated for 72 h. After incubation, some wells were processed immediately by detaching cells with trypsin and quantifying the number of cells using a haemocytometer (Day = 0). In the rest of the wells, the infection medium containing adenoviruses was removed and replaced by ECGM with supplements. After 72 h more of incubation, number of cells in these wells was determined using a haemocytometer as described above (Day = 3).

### Quantitative real-time PCR (qPCR)

Total RNA from HUVEC or HAoEC was extracted using the “Total RNA purification kit” (Norgen). For cDNA synthesis, 0.5 µg of total RNA were retrotranscribed in a final volume of 20 µl using the “High-capacity cDNA reverse transcription kit” (Applied Biosystems) according to the manufacturer’s recommendations. Samples were diluted by adding 80 µl of nuclease-free-water before qPCR analysis.

TaqMan qPCR reactions were set up by mixing 2.8 µl of cDNA reaction (5 ng/µl), 0.5 µl of the corresponding TaqMan Gene Expression Assay, and 5 µl of TaqMan Universal Master Mix II with UNG (2x) (ThermoFisher) in a final volume of 10 µl. Amplification took place in an Applied Biosystem 7500 fast real-time PCR system by incubation at 95 °C for 10 min, followed by 40 cycles of denaturation at 95 °C for 15 s and annealing/extension at 60 °C for 60 s. TaqMan gene expression assays used in this study were purchased from Applied Biosystems and are indicated in Supplementary Table S6.

Ct value in each sample was normalised according to the Ct value of the housekeeping gene *Hprt-1*. Analysis of qPCR data was carried out using the comparative 2^−ΔΔCt^ method.

### Gene array screening

Total RNA isolated from HUVEC transfected with miRNA “mimics” and stimulated with VEGF-A_165_ (50 ng/ml) for 1 h was retrotranscribed and used to analyse gene expression in a RT2 Profiler PCR Array Human Notch Signalling Pathway Plus Kit (Ref PAHS-059Y, Qiagen), or a RT2 Profiler PCR Array Human Cell Cycle Kit (Ref PAHS-020Z, Qiagen) following the manufacturer’s recommendations. cDNA used to analyse gene expression in a RT Profiler PCR Array Human Extracellular Matrix & Cell Adhesion Molecules Kit (Ref PAHS-013Z, Qiagen) was prepared from total RNA isolated from HUVEC transfected with miRNA “mimics” and stimulated with VEGF-A_165_ (50 ng/ml) for 4 h.

In all cases, PCR-based screening of gene array plates was performed under the following conditions: 10 min at 95, followed by 40 cycles of 15 s at 95 °C and 1 min at 60 °C. Data obtained from three independent experiments were analysed for statistically significant differences using the GeneGlobe Data Analysis Center (Qiagen). Genes showing a Log2(miR-133a-3p/miR-NC) > 1 and − Log10(*P* value) *P* ≤ 0.05 were selected as upregulated. Those with Log2(miR-133a-3p/miR-NC) <  − 1, and − Log10(*P* value) *P* ≤ 0.05 were selected as downregulated.

### Matrigel tube formation assay

HUVEC or HAoEC transfected with miRNA “mimics” were seeded onto Geltrex LDEV-Free Reduced Growth Factor Basement Membrane Matrix (ThermoFisher) in ECGM supplemented with 1% fetal calf serum (containing 50 ng/ml VEGF-A_165_ where indicated) in 96-well tissue culture plates at a density of 2 × 10^4^ cells/well. Tube formation was quantified after incubation at 37 °C for 24 h. The number of junctions was determined using ImageJ angiogenesis analyzer plugin software. Images were recorded using a Nikon DSFi1 digital camera mounted on a Nikon ECLIPSE TS100 microscope at 4× magnification.

### MTT assay

HUVEC transfected with miRNA “mimics” were seeded at a density of 4 × 10^3^ cells/well in ECGM containing supplement mixture, in 96-well tissue culture plates (without gelatin pre-coating). MTT assay was performed as described^48^, the morning after plating the cells (time = 0) and then after 3 days of incubation in ECGM with supplement mixture containing VEGF-A_165_ (50 ng/ml). Proliferation rate was calculated by dividing the absorbance at 540 nm of samples from cells incubated for 3 days by the absorbance of samples from cells at time = 0.

### Wound healing migration assay

7 × 10^4^/well HUVEC or HAoEC transfected with miRNA “mimics” were seeded in tissue culture plates from a “Cytoselect 24-well Wound Healing Assay Kit” (Cell Biolabs) following the manufacturer’s recommendations. Cell migration images were captured with a Nikon DSFi1 digital camera coupled to a Nikon ECLIPSE TS100 microscope at 4× magnification the morning after seeding (0 h) and 24 h later (24 h). The cell-free area was quantified with ImageJ software. The migrated area was calculated by subtracting the value of the non-migrated (cell-free area at time 24 h) from the initial wound area (cell-free area at time 0 h), and then expressing this value as a percentage of wound area at time zero.

When indicated 2 mM (final concentration) hydroxyurea was added to the medium to inhibit cell proliferation during the 24 h incubation period.

### Flow cytometry analysis of cell cycle progression

HUVEC transfected with miR mimics miR-NC (negative control) or miR-133a-3p as described, were fixed in 70% ethanol at 4 °C for 16 h. Fixed cells were washed twice with PBS 1 × to remove ethanol traces and resuspended in 300 µl propidium iodide (50 µg/ml) and incubated at room temperature for 15 min in the dark. The percentage of cells in each phase of the cell cycle was determined by flow cytometry using a BD FACSMelody flow cytometer.

### Western blot analysis

HUVEC (3 × 10^5^ cells/well) were plated in 6-well tissue culture plates and transfected with a mimic for human miR-133a-3p or a miR mimic control as indicated. Transfected cells were left unstimulated or stimulated with VEGF-A_165_ (50 ng/ml) for the indicated times. Total proteins were isolated by direct cell lyses in NuPAGE LDS Sample Buffer (Life Technology, UK) containing 0.05% beta-Mercaptoethanol. Samples were heated at 100 °C for 10 min to solubilise DNA and maximise protein denaturation, and subsequently cooled on ice before storage at − 20 °C until further use.

Western blot membranes were incubated with a 1:2000 dilution of a rabbit polyclonal anti-cyclin B1 antibody (H-433) (Santa Cruz Biotechnology), a 1:1000 dilution of a mouse monoclonal (156-3C11) anti-CD44 antibody (Cell Signalling), a 1:1000 dilution of a rabbit monoclonal (19A10) anti-phospho-VEGF Receptor 2 (Tyr1175) (Cell Signalling), 1:2000 dilution of a rabbit monoclonal (55B11) anti-VEGF Receptor 2 antibody (Cell Signalling), a 1:1000 dilution of a rabbit monoclonal (197G2) anti-Phospho-p44/42 MAPK (Erk1/2) (Thr202/Tyr204) antibody (Cell Signalling), a 1:1000 dilution of a rabbit anti-p44/42 MAPK (Erk1/2) antibody (Cell Signalling), a 1:1000 dilution of a rabbit anti-DSCR1 antibody (Sigma) (for RCAN1.4 detection), or a 1:3000 dilution of a mouse monoclonal anti-tubulin antibody (Sigma). Primary antibodies were detected by incubation in either a 1:5000 dilution of peroxidase-conjugated sheep anti-mouse immunoglobuilin antibody (Sigma) or 1:5000 dilution of peroxidase-conjugated donkey anti-rabbit immunoglobulin antibody (GE Healthcare) depending on the origin of the primary antibody. All antibody dilutions were carried out in TBS-T.

### Bioinformatic analysis

miR-133a-3p and -5p target site prediction in the selected genes was analysed using miRWalk^49^, Target scan^50^, and miRDB^51^ data bases.

Ingenuity Pathway Analysis (IPA, Qiagen)^52^ was used to perform functional enrichment analyses on the genes identified as up- or downregulated after gene array screening. Enrichment results with Benjamini–Hochberg adjusted *P* value < 0.05 were considered significant. Circular plots, summarizing the relation between selected pathways or functions and the associated up- or downregulated genes were generated with the R package GOplot^53^.

### Statistical analysis

Results are shown as mean ± SE. The significance of the differences between two groups was analysed by unpaired, two-tailed Student's *t*-test. ANOVA with post hoc Tukey's comparison test was used for comparison among more than 2 groups. Differences were considered statistically significant at *P* ≤ 0.05.

## Supplementary Information

Supplementary Information.

## Data Availability

All data generated or analysed during this study are included in this published article (and its Supplementary Information file).
