# Supplementary material for: Aberrant expression of miR-133a in endothelial cells inhibits angiogenesis by reducing pro-angiogenic but increasing anti-angiogenic gene expression
Source: Sci Rep. 2022 Aug 30;12:14730. doi: 10.1038/s41598-022-19172-x (PMC9427859; doi:10.1038/s41598-022-19172-x)
Supplement: Supplementary file 1 — Supplementary Information. [file 41598_2022_19172_MOESM1_ESM.pdf]

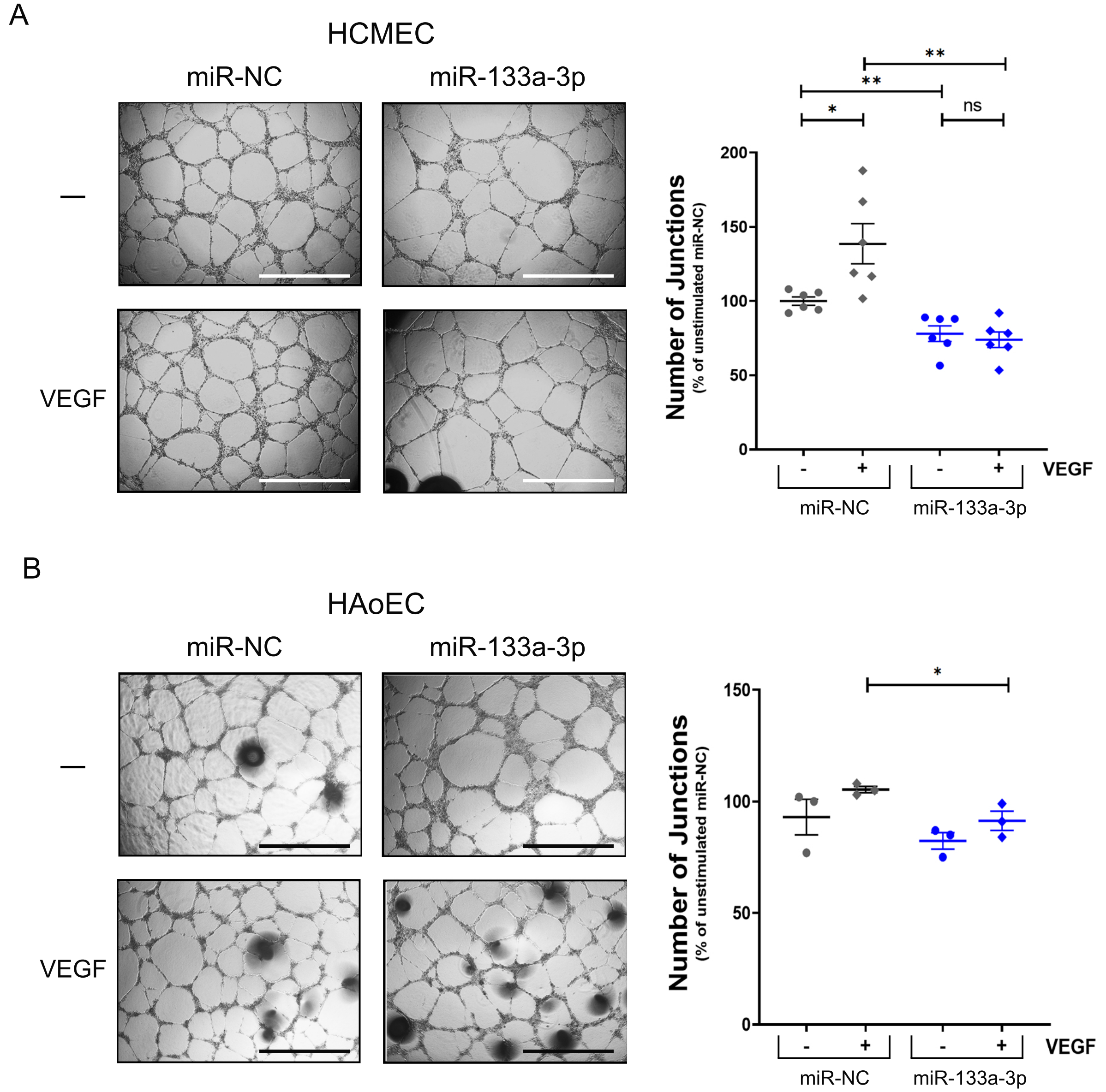

Figure S1. Ectopic expression of miR-133a-3p in HCMEC or HAoEC inhibits VEGF-induced angiogenesis. Human Cardiac Microvascular Endothelial Cells (HCMEC) (A) or Human Aortic Endothelial Cells (HAoEC) (B) transfected with miR-133a-3p mimics or a negative control (miR-NC) were seeded on Growth-Factor Reduced Matrigel (Geltrex) in ECGM medium containing 1% FBS and were left untreated (-) or were stimulated with VEGF-A165 at 50 ng/ml (+) as indicated. Images show representative fields from experiments quantified in the histogram. Data are shown as mean ± SE, n = 6 (A) or n = 3 (B). ns = non-significant; *, $P \leq 0.05$; **, $P \leq 0.01$ (unpaired two-tailed Student's *t*-test when comparing the indicated groups). Scale bars, 1000 µm. Image J for windows bundled with 64-bit Java 1.8.0_172 was used in this figure (https://imagej.nih.gov/ij/).

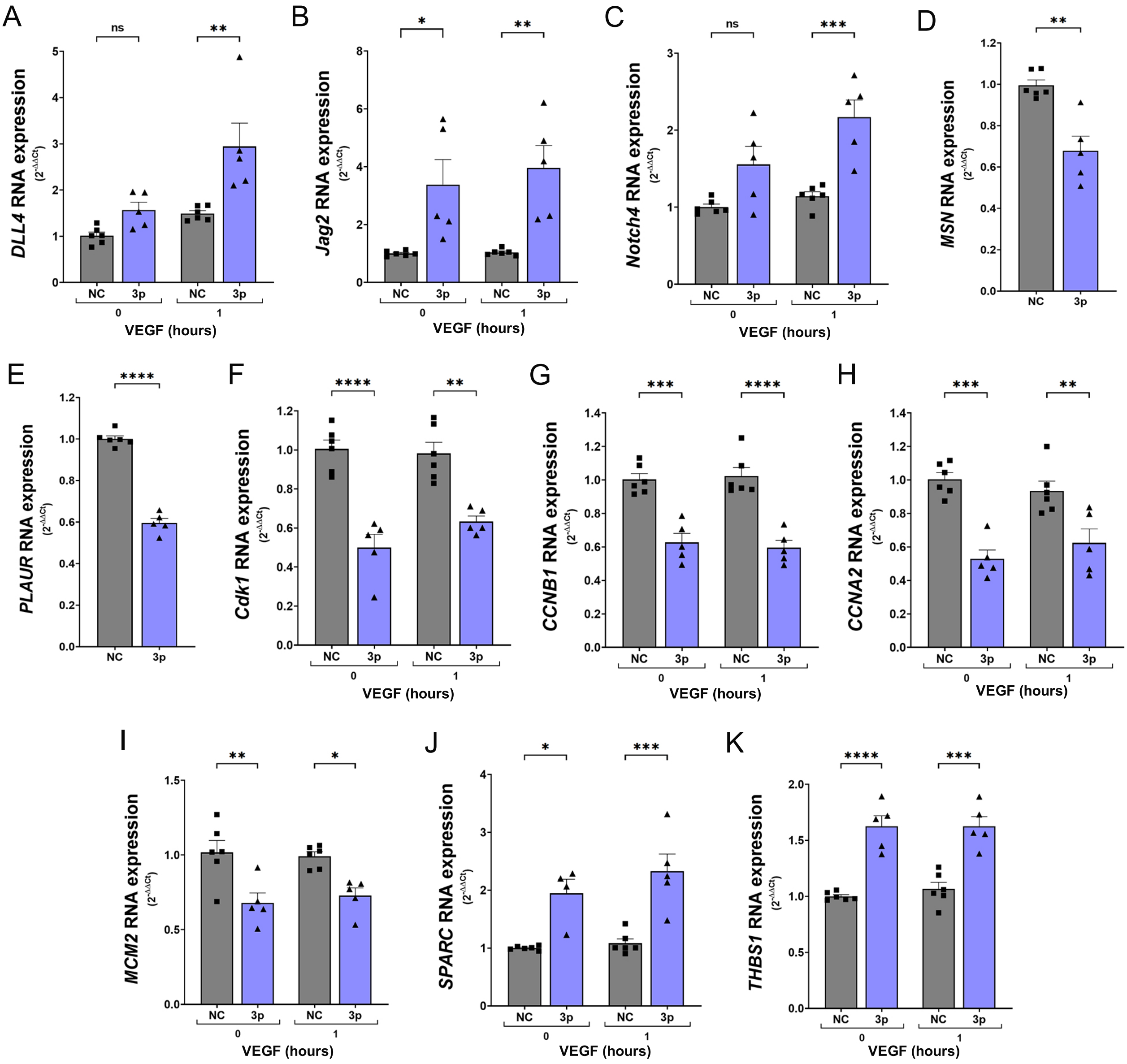

Figure S2. Aberrant expression of miR-133a-3p in primary Human Aortic Endothelial Cells (HAoEC) significantly alters the expression of genes encoding components of Notch signalling, cell migration, cell cycle regulators, and extracellular matrix. HAoEC were transfected with a control miR mimic (NC, grey bars) or a mimic for human miR-133a-3p (3p, blue bars) and RNA levels for the indicated genes were determined by qPCR. Expression of genes encoding Notch-signalling components (A-C), motility proteins (D, E), cell-cyle regulators (F-I) or extracellular matrix proteins (J-K) were significantly altered in cells expressing miR-133a-3p. Data are shown as mean ± SE, n = 5-6. Data were analysed for statistical differences by two-way ANOVA with *post hoc* Tukey's comparison test.
ns = non-significant; *, $P \leq 0.05$; **, $P \leq 0.01$; ***, $P \leq 0.001$; ****, $P \leq 0.0001$.
VEGF = VEGF-A165.

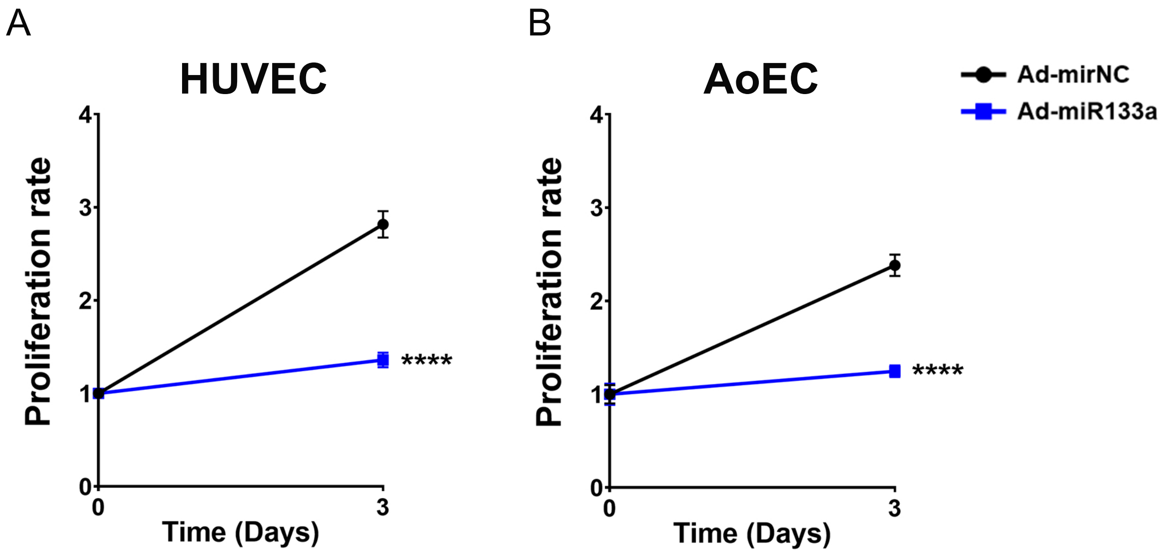

Figure S3. Ectopic expression of miR-133a in HUVEC or AoEC significantly inhibits endothelial cell proliferation. HUVEC (A) or HAoEC (B) infected with adenovirus negative control (AdmiR-NC, black line) or with an adenovirus expressing miR-133a (AdmiR-133a, blue line) were cultured in ECGM containing VEGF-A165 (50 ng/ml) for the indicated times. Number of cells were determined at the indicated time by detachment with trypsine and cell counting using a heamocytometer. Proliferation rate was calculated as number of cells after 3 days of culture divided by number of cells at 0 days. Data are shown as mean ± SE, n = 5. ****, P ≤ 0.0001 AdmiR-133a *vs* AdmiR-NC at 3 Days analysed by two-way ANOVA with *post hoc* Bonferroni's comparison test.

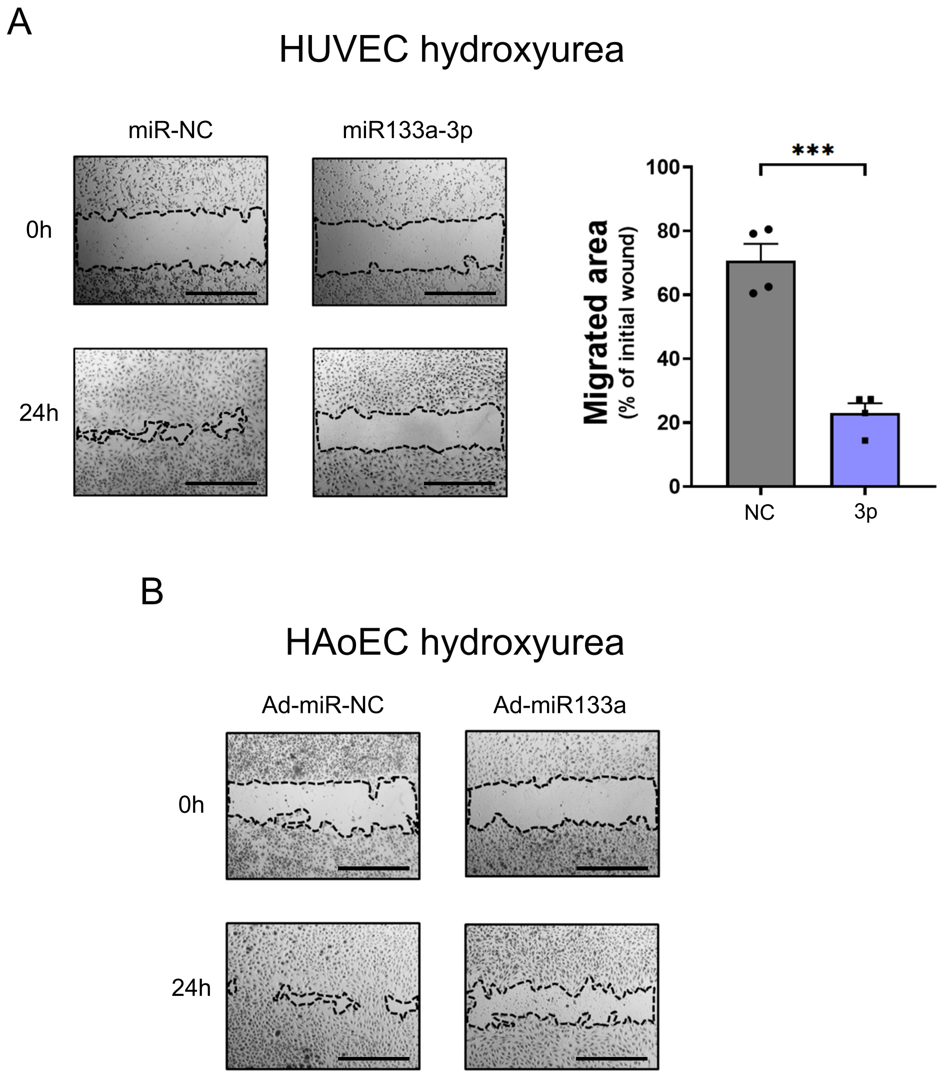

Figure S4. miR-133a inhibits HUVEC and HAoEC migration in the presence of the proliferation inhibitor hydroxyurea. (A) Representative images of wound-healing migration assays performed with HUVEC transfected with microRNA mimics for miR-133a-3p, or a negative control (miR-NC). Images were taken at time zero (0h) and after incubation for 24 hours in endothelial cell medium in the presence of 2 mM hydroxyurea (24h).The migrated area was calculated by subtracting the value of the non-migrated area from the wound area at time zero and expressing this as a percentage of the total area at time zero. Scale bars, 1000 μm. Data are shown as mean ± SE, n = 4. Data were analysed for statistical differences by one-way ANOVA with *post hoc* Tukey's comparison test. ***, $P \leq 0.001$. (B) Human Aortic Endothelial Cells were infected with control adenovirus (Ad-miR-NC) or an adenovirus expressing miR-133a. (Ad-miR-133a) at MOI=200 for 72 hours. Motility of the infected cells was analysed in wound-healing migration assays as described in (A). Images are representative of two independent experiments. Scale bars, 1000 μm. Image J for windows bundled with 64-bit Java 1.8.0_172 was used in this figure (https://imagej.nih.gov/ij/).

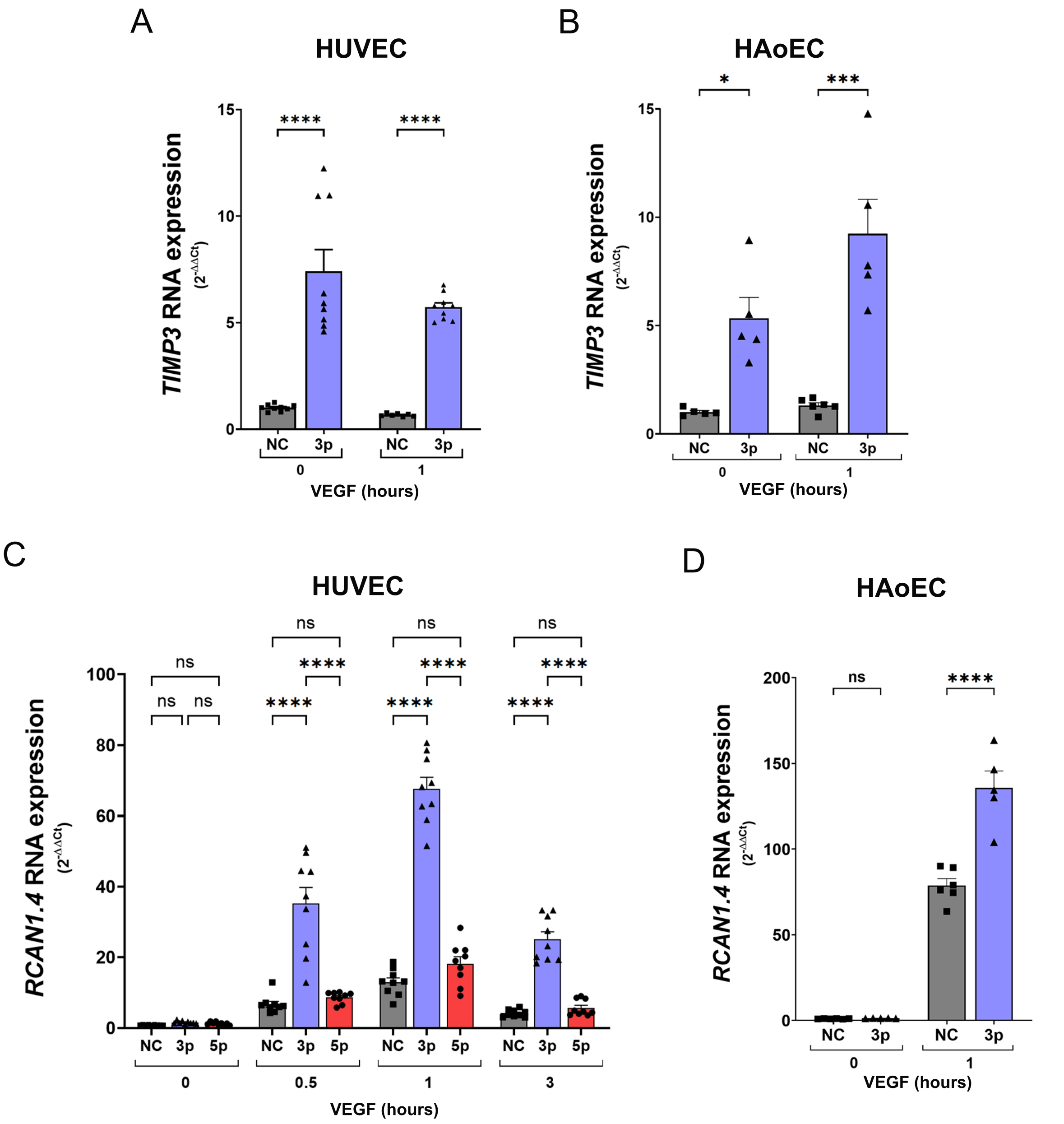

Figure S5. Changes in gene expression of *TIMP3* and *RCAN1.4* in endothelial cells expressing miR-133a. HUVEC (A, C) or HAoEC (B, D) transfected with miR-133a-3p (blue columns), miR-133a-5p (red columns), or negative control miR-NC (grey columns) were left unstimulated (0h) or were stimulated with VEGF-A165 as indicated. RNA levels of *TIMP3* (A, B) and *RCAN1.4* (C, D) significantly increased in miR-133a-3p transfected cells. Ct values were normalised using the Ct values for the housekeeping gene *Hprt-1*. Analysis of data was carried out using the comparative 2^−ΔΔCt method referred to miR-NC (0h). Data are shown as mean ± SE, n = 7 in HUVEC or n = 5 in AoEC. ns = non-significant; ****, $P \leq 0.0001$; ***, $P \leq 0.001$ two-way ANOVA with *post hoc* Tukey's comparison test for the indicated groups.

Original blots used in figure 7A

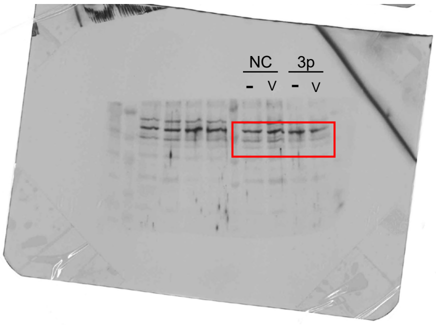

Original Blot corresponding to the analysis of Cyclin B1 in Figure 7A. The part of the blot presented in Fig 7A is marked with a red rectangle

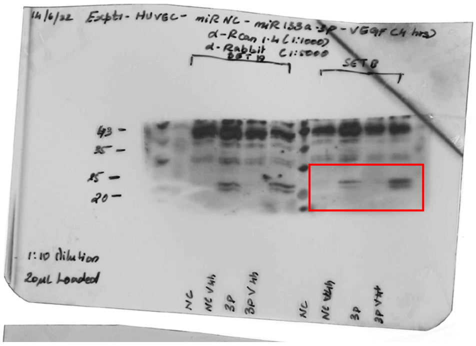

Original Blot corresponding to the analysis of RCAN1.4 in Figure 7A. The part of the blot presented in Fig 7A is marked with a red rectangle

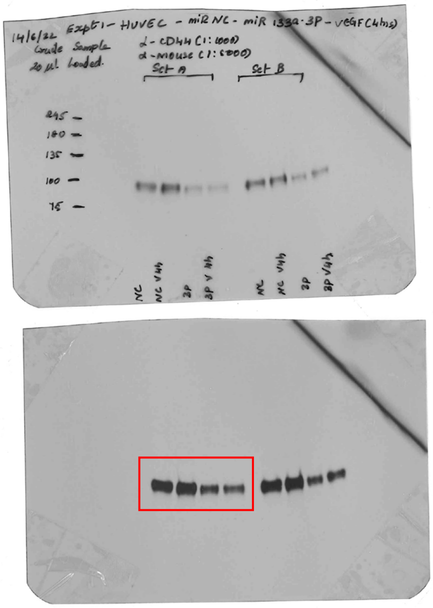

Original Blot corresponding to the analysis of CD44 in Figure 7A. The part of the blot presented in Fig 7A is marked with a red rectangle. Another exposure of the same blot showing the protein ladder markers is also provided.

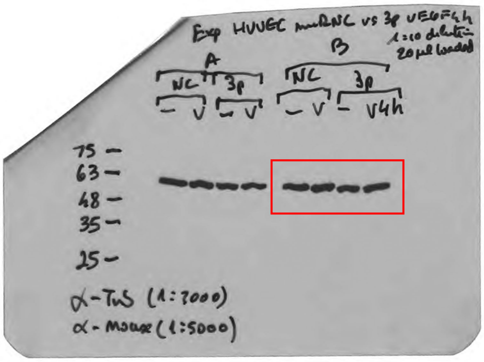

Original Blot corresponding to the analysis of Tubulin in Figure 7A. The part of the blot presented in Fig 7A is marked with a red rectangle

Original blots used in figure 7B

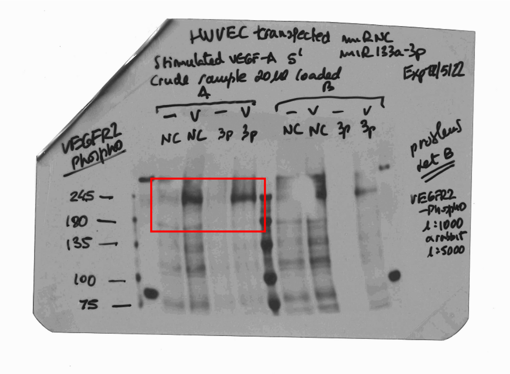

Original Blot corresponding to the analysis of Phospho-VEGFR2 in Figure 7B. The part of the blot presented in Fig 7B is marked with a red rectangle

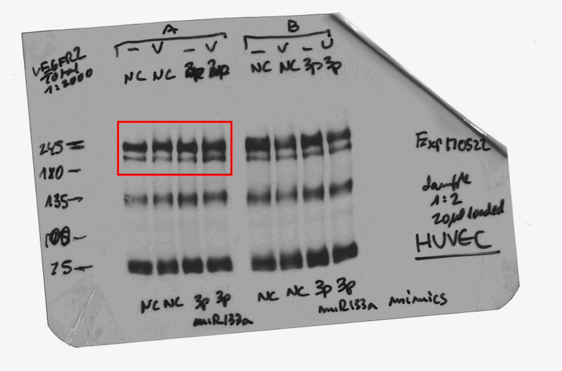

Original Blot corresponding to the analysis of Total-VEGFR2 in Figure 7B. The part of the blot presented in Fig 7B is marked with a red rectangle

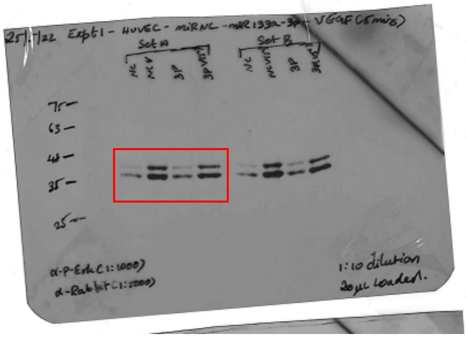

Original Blot corresponding to the analysis of Phospho-Erk in Figure 7B. The part of the blot presented in Fig 7B is marked with a red rectangle

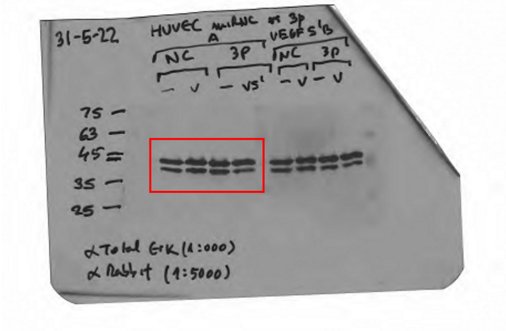

Original Blot corresponding to the analysis of Total-Erk in Figure 7B. The part of the blot presented in Fig 7B is marked with a red rectangle

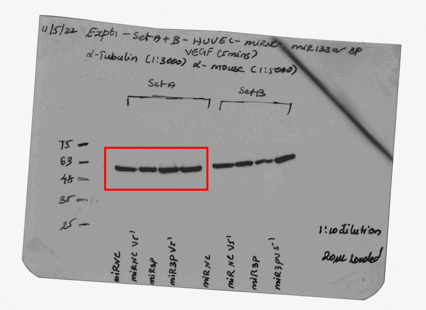

Original Blot corresponding to the analysis of Tubulin in Figure 7B. The part of the blot presented in Fig 7B is marked with a red rectangle

Position	Gene Symbol	AVG ΔC_t		$2^{-\Delta C_t}$		Fold Change	p-value
		miR133a-3p VEGF 1h	miR-NC VEGF 1h	miR133a-3p VEGF 1h	miR-NC VEGF 1h	miR133a-3p VEGF 1h/miR-NC VEGF 1h	miR133a-3p VEGF 1h / miR-NC VEGF 1h
A01	ADAM10	2.37	2.87	0.19382	0.136999	1.41	0.010688
A02	ADAM17	4.33	4.38	0.049602	0.047931	1.03	0.967439
A03	AES	2.39	2.54	0.191036	0.172458	1.11	0.573270
A04	AXIN1	6.09	6.06	0.014722	0.01497	0.98	0.883499
A05	CBL	6	6.03	0.015596	0.015344	1.02	0.862729
A06	CCND1	3.04	2.06	0.121559	0.23953	0.51	0.032068
A07	CCNE1	7.54	6.74	0.005356	0.009343	0.57	0.003667
A08	CD44	4.81	3.01	0.035679	0.124391	0.29	0.002287
A09	CDKN1A	1.98	2.12	0.254345	0.229741	1.11	0.622333
A10	CFLAR	3.68	4.29	0.078231	0.051042	1.53	0.001997
A11	CHUK	4.52	5.16	0.043592	0.028058	1.55	0.474799
A12	CTNNB1	2.52	3.11	0.174498	0.116168	1.5	0.165019
B01	DLL1	7.63	7.76	0.005062	0.004599	1.1	0.868859
B02	DLL3	13.89	14.94	0.000066	0.000032	2.08	0.500788
B03	DLL4	3.54	6.41	0.086118	0.01174	7.34	0.002103
B04	EP300	5.11	5.08	0.028991	0.029642	0.98	0.744398
B05	ERBB2	6	6.48	0.015619	0.011181	1.4	0.084305
B06	FIGF	12.68	12.13	0.000152	0.000224	0.68	0.427723
B07	FOS	7.3	7.76	0.006344	0.004614	1.37	0.312468
B08	FOSL1	3.97	3.84	0.063655	0.069966	0.91	0.347284
B09	FZD7	11.95	12.28	0.000253	0.000201	1.26	0.608977
B10	GLI1	11.23	12.12	0.000415	0.000225	1.85	0.430816
B11	GSK3B	4.14	4.15	0.056579	0.056255	1.01	0.985468
B12	HDAC1	3.91	4.37	0.066313	0.04834	1.37	0.042302
C01	HES1	4.06	3.9	0.059933	0.066897	0.9	0.748074
C02	HEY1	6.71	8.12	0.009553	0.003606	2.65	0.003644
C03	HOXB4	8.25	8.04	0.003276	0.00381	0.86	0.663624
C04	HR	11.48	13.02	0.00035	0.000121	2.9	0.280384
C05	ID1	6.15	5.02	0.014127	0.030772	0.46	0.011025
C06	IFNG	15.93	16.78	0.000016	0.000009	1.81	0.333035
C07	IL2RA	15.83	16.69	0.000017	0.000009	1.81	0.358301
C08	JAG1	4.13	5.18	0.056924	0.027556	2.07	0.060391
C09	JAG2	4.1	5.45	0.058398	0.022886	2.55	0.008049
C10	KRT1	16.16	16.78	0.000014	0.000009	1.55	0.308032
C11	LFNG	6.84	6.92	0.00875	0.008239	1.06	0.840951
C12	LMO2	2.91	3.61	0.133194	0.081731	1.63	0.038285
D01	LOR	16.15	16.31	0.000014	0.000012	1.12	0.688941
D02	LRP5	7.55	7.59	0.005347	0.00519	1.03	0.851724
D03	MAML1	6.31	5.87	0.012626	0.017067	0.74	0.198066
D04	MAML2	6.44	6.51	0.011547	0.010951	1.05	0.889567
D05	MFNG	3.07	2.83	0.119359	0.140923	0.85	0.021107
D06	MMP7	8.32	8.22	0.003136	0.003351	0.94	0.664009
D07	NCOR2	4.53	4.27	0.04326	0.052003	0.83	0.495544
D08	NCSTN	3.27	3.61	0.103865	0.081666	1.27	0.000225
D09	NFKB1	5.71	6.01	0.01913	0.015483	1.24	0.441816
D10	NFKB2	5.4	5.68	0.023755	0.019526	1.22	0.387127
D11	NOTCH1	5.99	6.37	0.015696	0.012083	1.3	0.521508
D12	NOTCH2	6.14	5.67	0.014193	0.019697	0.72	0.141066
E01	NOTCH3	9.74	9.88	0.001168	0.001064	1.1	0.905228
E02	NOTCH4	4.5	5.81	0.044197	0.017865	2.47	0.011842
E03	NUMB	4.29	4.21	0.051166	0.054054	0.95	0.124417
E04	PAX5	15.62	16.36	0.00002	0.000012	1.67	0.426859
E05	POFUT1	7.7	7.9	0.0048	0.004199	1.14	0.366457
E06	PPARG	8.36	10.18	0.003044	0.000865	3.52	0.000338
E07	PSEN1	4.63	4.42	0.040491	0.046872	0.86	0.032367
E08	PSEN2	6	6.02	0.015611	0.0154	1.01	0.928805
E09	PSENEN	4.33	4.4	0.049687	0.047374	1.05	0.620692
E10	PTCRA	14.15	15.3	0.000055	0.000025	2.22	0.400245
E11	RBPJL	15.59	15.66	0.00002	0.000019	1.05	0.763113
E12	RFNG	5.21	5.44	0.027062	0.023039	1.17	0.088466
F01	RUNX1	6.81	6.06	0.008936	0.014977	0.6	0.033440
F02	SEL1L	3.35	3.84	0.097817	0.069881	1.4	0.006913
F03	SHH	11.97	11.35	0.00025	0.000382	0.65	0.007158
F04	SMO	7.62	7.37	0.005075	0.00603	0.84	0.105797
F05	SNW1	5.2	4.82	0.027124	0.035378	0.77	0.170943
F06	STAT6	4.16	3.93	0.055907	0.065676	0.85	0.108075
F07	TLE1	4.7	4.75	0.03838	0.037252	1.03	0.761570
F08	WISP1	12.63	15.4	0.000158	0.000023	6.83	0.000754
F09	ADA	4.92	4.89	0.033064	0.033743	0.98	0.912166
F10	AFAP1L2	9.3	12.01	0.001592	0.000242	6.57	0.056412
F11	DTX1	15.26	16.07	0.000025	0.000015	1.75	0.428849
F12	H19	11.49	13.47	0.000347	0.000088	3.93	0.032924
G01	Hes4	4.95	6.64	0.032401	0.010001	3.24	0.044112
G02	HES5	13.99	15.62	0.000062	0.00002	3.11	0.407130
G03	HEY2	10.02	10.17	0.000966	0.000868	1.11	0.872676
G04	HEYL	16	16.68	0.000015	0.00001	1.6	0.230238
G05	NRARP	5.7	7.11	0.019182	0.007259	2.64	0.046670
G06	SCGB1A1	15.64	16.78	0.00002	0.000009	2.22	0.048320
G07	SERPINA3	13.27	13.9	0.000101	0.000065	1.55	0.348460
G08	SLC6A12	16.66	16.78	0.00001	0.000009	1.09	0.629893
G09	SNAI2	10.11	9.59	0.000902	0.001301	0.69	0.321168
G10	TFF1	16.3	16.58	0.000012	0.00001	1.21	0.481258
G11	TNFSF10	4.19	4.23	0.054855	0.053357	1.03	0.877834
G12	UBD	10.53	10.94	0.000678	0.00051	1.33	0.547084
H01	ACTB	-2.73	-2.65	6.644773	6.274732	1.06	0.419120
H02	B2M	-0.76	-0.36	1.698719	1.28043	1.33	0.182271
H03	GAPDH	-1.15	-1.33	2.222983	2.508923	0.89	0.269595
H04	HPRT1	5.96	5.59	0.016051	0.020728	0.77	0.007666
H05	RPLP0	-1.31	-1.26	2.48292	2.393297	1.04	0.704859

Supplementary Table S1. Differential gene expression analysis of RT2 Profiler PCR Array Human Notch Signalling Pathway Plus Kit. HUVEC transfected with miR-133a-3p or negative control miR-NC were stimulated with VEGF-A165 for 1 hour. Upregulated and downregulated genes are highlighted in red and green, respectively. Genes showing values of AVG ΔCt ≥ 10 indicating high number of amplification cycles required in the PCR analysis were not considered in further experiments to improve accuracy and reproducibility of data.

Position	Gene Symbol	AVG ΔC_t		2^-ΔC_t		Fold Change	p-value
		miR133a-3p VEGF 1h	miR-NC VEGF 1h	miR133a-3p VEGF 1h	miR-NC VEGF 1h	miR133a-3p VEGF 1h/miR-NC VEGF 1h	miR133a-3p VEGF 1h / miR-NC VEGF 1h
A01	ABL1	5.34	6.03	0.024615	0.015268	1.61	0.370162
A02	ANAPC2	6.51	7.12	0.011003	0.007179	1.53	0.163561
A03	ATM	7.71	7.98	0.004776	0.003964	1.2	0.872675
A04	ATR	8.28	8.4	0.003216	0.002964	1.08	0.764296
A05	AURKA	6.76	5.73	0.009201	0.018801	0.49	0.193957
A06	AURKB	9.17	7.13	0.001741	0.007153	0.24	0.117954
A07	BCCIP	4.74	4.44	0.037322	0.046224	0.81	0.358242
A08	BCL2	8.81	10.45	0.002229	0.000715	3.12	0.002742
A09	BIRC5	12.19	10.18	0.000215	0.000863	0.25	0.113655
A10	BRCA1	10.06	8.42	0.000936	0.00291	0.32	0.128632
A11	BRCA2	9.49	9.05	0.001395	0.001889	0.74	0.518049
A12	CASP3	4.08	4.58	0.05904	0.041737	1.41	0.568454
B01	CCNA2	7.19	4.63	0.006844	0.040488	0.17	0.007077
B02	CCNB1	6.88	4.77	0.008502	0.036538	0.23	0.014929
B03	CCNB2	8.04	5.97	0.003788	0.015924	0.24	0.101765
B04	CCNC	6.14	6.9	0.014182	0.008399	1.69	0.310672
B05	CCND1	2.69	2.97	0.155198	0.127381	1.22	0.756967
B06	CCND2	6.84	8.46	0.008759	0.00283	3.09	0.280935
B07	CCND3	3.61	5.03	0.082101	0.030607	2.68	0.029723
B08	CCNE1	7.52	7.47	0.005459	0.005632	0.97	0.907534
B09	CCNF	7.33	7.27	0.006214	0.006487	0.96	0.575306
B10	CCNG1	1.48	3.26	0.358697	0.104205	3.44	0.037099
B11	CCNG2	5.08	5.59	0.029486	0.020746	1.42	0.704174
B12	CCNH	6.44	7.4	0.011521	0.00594	1.94	0.576288
C01	CCNT1	6.45	7.22	0.011417	0.006717	1.7	0.805782
C02	CDC16	4.48	5.2	0.044827	0.027234	1.65	0.533739
C03	CDC20	8.37	6.42	0.003019	0.011709	0.26	0.08117
C04	CDC25A	8.32	8.1	0.00313	0.003657	0.86	0.57382
C05	CDC25C	12.28	10.35	0.000201	0.000764	0.26	0.286847
C06	CDC34	5.74	6.49	0.018661	0.011092	1.68	0.240041
C07	CDC6	7.66	6.54	0.004951	0.010772	0.46	0.223849
C08	CDK1	6.57	3.44	0.010508	0.0921	0.11	0.000561
C09	CDK2	6.22	6.2	0.013418	0.013595	0.99	0.663534
C10	CDK4	4.09	4.72	0.058805	0.038073	1.54	0.545356
C11	CDK5R1	9.49	9.87	0.001387	0.001067	1.3	0.530816
C12	CDK5RAP1	9.72	10.39	0.001187	0.000743	1.6	0.596426
D01	CDK6	4.61	6.94	0.04084	0.008172	5	0.067356
D02	CDK7	5.02	5.13	0.030758	0.028553	1.08	0.98645
D03	CDK8	7.4	7.42	0.005916	0.005848	1.01	0.912495
D04	CDKN1A	2.25	3.54	0.209834	0.085955	2.44	0.157594
D05	CDKN1B	4.96	5.77	0.032056	0.018345	1.75	0.489002
D06	CDKN2A	11.48	12.12	0.000349	0.000224	1.55	0.482616
D07	CDKN2B	7.18	8.71	0.00689	0.002394	2.88	0.094351
D08	CDKN3	7.19	6.41	0.006868	0.011727	0.59	0.33057
D09	CHEK1	7.44	6.66	0.00574	0.009899	0.58	0.283328
D10	CHEK2	8.09	8.63	0.003657	0.00253	1.45	0.432738
D11	CKS1B	6.14	4.94	0.014219	0.032558	0.44	0.159196
D12	CKS2	4.19	4.44	0.054895	0.046006	1.19	0.766768
E01	CUL1	5.12	5.19	0.02882	0.027368	1.05	0.918052
E02	CUL2	5.1	5.55	0.029246	0.02138	1.37	0.205873
E03	CUL3	3.82	4.62	0.070815	0.040534	1.75	0.448347
E04	E2F1	8.13	7.39	0.003577	0.005964	0.6	0.307408
E05	E2F4	4.36	5.41	0.048604	0.023558	2.06	0.501406
E06	GADD45A	6.33	5.55	0.012446	0.021391	0.58	0.133284
E07	GTSE1	8.37	6.45	0.003024	0.011404	0.27	0.087604
E08	HUS1	5.28	6.23	0.025811	0.013311	1.94	0.541133
E09	KNTC1	8.79	8.1	0.002266	0.003652	0.62	0.428603
E10	KPNA2	2.09	2.75	0.234331	0.148985	1.57	0.794139
E11	MAD2L1	7.9	6.16	0.004173	0.014016	0.3	0.130544
E12	MAD2L2	9.12	8.91	0.001795	0.002077	0.86	0.564985
F01	MCM2	7.9	6.7	0.004176	0.009641	0.43	0.050139
F02	MCM3	6.32	5.57	0.012524	0.021059	0.59	0.182968
F03	MCM4	7.4	6.29	0.005925	0.012751	0.46	0.007047
F04	MCM5	6.28	5.84	0.012886	0.017422	0.74	0.579795
F05	MDM2	2.68	3.82	0.155521	0.070568	2.2	0.104164
F06	MKI67	7	5.16	0.007788	0.0279	0.28	0.245959
F07	MNAT1	6.4	7.08	0.011809	0.007386	1.6	0.551554
F08	MRE11A	6.51	6.8	0.010994	0.008984	1.22	0.966266
F09	NBN	4.84	5.69	0.034921	0.019365	1.8	0.092656
F10	RAD1	6.49	7.16	0.01111	0.006969	1.59	0.476448
F11	RAD17	6.4	7.3	0.011843	0.006341	1.87	0.241411
F12	RAD51	9.4	8.35	0.001476	0.003064	0.48	0.238363
G01	RAD9A	8.4	8.51	0.002964	0.002752	1.08	0.936191
G02	RB1	5.49	5.56	0.022225	0.021268	1.05	0.933481
G03	RBBP8	8.25	8.15	0.00329	0.003516	0.94	0.936822
G04	RBL1	7.71	7.21	0.004772	0.006754	0.71	0.415562
G05	RBL2	5.35	5.93	0.024592	0.016415	1.5	0.548759
G06	SERTAD1	6.5	7.16	0.011049	0.006976	1.58	0.267238
G07	SKP2	7.09	7.14	0.007348	0.007102	1.03	0.73783
G08	STMN1	2.5	1.52	0.176889	0.348554	0.51	0.032378
G09	TFDP1	4.22	3.63	0.053694	0.080782	0.66	0.455342
G10	TFDP2	7.18	7.67	0.006887	0.004909	1.4	0.922733
G11	TP53	3.04	3.74	0.121287	0.074857	1.62	0.614892
G12	WEE1	5.01	5.49	0.0311	0.022296	1.39	0.373908
H01	ACTB	-2.49	-2.53	5.637138	5.76735	0.98	0.847387
H02	B2M	-0.12	0.22	1.087523	0.8606	1.26	0.432388
H03	GAPDH	-1.18	-1.71	2.269593	3.260933	0.7	0.385742
H04	HPRT1	4.65	4.97	0.039753	0.032014	1.24	0.485624
H05	RPLP0	-0.85	-0.95	1.807953	1.929933	0.94	0.734311

Supplementary Table S2. Differential gene expression analysis of RT2 Profiler PCR Array Human Cell Cycle Kit. HUVEC transfected with miR-133a-3p or negative control miR-NC were stimulated with VEGF-A165 for 1 hour. Upregulated and downregulated genes are highlighted in red and green, respectively. Genes showing values of AVG ΔCt ≥ 10 indicating high number of amplification cycles required in the PCR analysis were not considered in further experiments to improve accuracy and reproducibility of data.

Position	Gene Symbol	AVG ΔC_t		2^-ΔC_t		Fold Change	p-value
		miR133a-3p VEGF 1h	miR-NC VEGF 1h	miR133a-3p VEGF 1h	miR-NC VEGF 1h	miR133a-3p VEGF 1h/miR-NC VEGF 1h	miR133a-3p VEGF 1h / miR-NC VEGF 1h
A01	ADAMTS1	3.51	4.63	0.087613	0.040452	2.17	0.009728
A02	ADAMTS13	12.76	13.14	0.000145	0.000111	1.30	0.425539
A03	ADAMTS8	16.85	16.41	0.000008	0.000011	0.73	0.557347
A04	CD44	3.12	2.32	0.115321	0.200883	0.57	0.032911
A05	CDH1	16.85	17.27	0.000008	0.000006	1.33	0.752087
A06	CLEC3B	13.88	13.13	0.000067	0.000112	0.60	0.642833
A07	CNTN1	14.66	13.55	0.000039	0.000084	0.46	0.347898
A08	COL11A1	14.94	16.46	0.000032	0.000011	2.87	0.291711
A09	COL12A1	6.17	7.21	0.013842	0.006753	2.05	0.153185
A10	COL14A1	12.18	11.44	0.000215	0.000359	0.60	0.288410
A11	COL15A1	14.64	15.9	0.000039	0.000016	2.40	0.472659
A12	COL16A1	11.86	11.62	0.000269	0.000318	0.85	0.625143
B01	COL1A1	14.86	12.47	0.000034	0.000176	0.19	0.054788
B02	COL4A2	0.35	1.17	0.785597	0.44353	1.77	0.185808
B03	COL5A1	2.22	3.33	0.214854	0.099275	2.16	0.141926
B04	COL6A1	5.58	6.02	0.020913	0.015454	1.35	0.467034
B05	COL6A2	10.59	10.13	0.000651	0.000892	0.73	0.975068
B06	COL7A1	14.68	14.35	0.000038	0.000048	0.80	0.765503
B07	COL8A1	4.25	3.45	0.05246	0.09175	0.57	0.521567
B08	CTGF	-0.35	-0.8	1.272852	1.735088	0.73	0.601233
B09	CTNNA1	2.02	1.99	0.245921	0.251472	0.98	0.961821
B10	CTNNB1	2.57	3.55	0.168494	0.085373	1.97	0.024149
B11	CTNND1	3.42	3	0.093283	0.125032	0.75	0.264605
B12	CTNND2	16.8	15.62	0.000009	0.00002	0.44	0.336888
C01	ECM1	9.44	9.05	0.001437	0.001885	0.76	0.640300
C02	FN1	-2.43	-1.94	5.375817	3.837224	1.40	0.256214
C03	HAS1	13.49	13.91	0.000087	0.000065	1.34	0.941511
C04	ICAM1	3.17	4	0.111119	0.062604	1.77	0.116271
C05	ITGA1	7.13	8.1	0.00716	0.003652	1.96	0.251296
C06	ITGA2	3.84	3.48	0.069607	0.089804	0.78	0.014392
C07	ITGA3	3.25	2.89	0.105097	0.134514	0.78	0.497416
C08	ITGA4	6.49	7.9	0.011127	0.004184	2.66	0.345427
C09	ITGA5	0.44	0.9	0.739286	0.535757	1.38	0.188920
C10	ITGA6	2.1	3.27	0.232914	0.103672	2.25	0.004711
C11	ITGA7	13.45	13.93	0.00009	0.000064	1.40	0.191173
C12	ITGA8	11.79	15.66	0.000282	0.000019	14.60	0.001639
D01	ITGAL	15.52	16.38	0.000021	0.000012	1.81	0.231277
D02	ITGAM	16.59	16.47	0.00001	0.000011	0.92	0.850826
D03	ITGAV	1.72	2.31	0.302769	0.202065	1.50	0.097940
D04	ITGB1	-0.32	-0.06	1.250113	1.039281	1.20	0.095719
D05	ITGB2	10.98	12.45	0.000495	0.000179	2.76	0.153679
D06	ITGB3	4.55	4.41	0.042643	0.046962	0.91	0.731497
D07	ITGB4	6.89	6.58	0.008432	0.010462	0.81	0.652303
D08	ITGB5	2.54	3.01	0.171767	0.123782	1.39	0.366620
D09	ANOS1	14.11	12.67	0.000056	0.000154	0.37	0.124574
D10	LAMA1	15.86	17.27	0.000017	0.000006	2.65	0.229367
D11	LAMA2	9.66	10.19	0.001232	0.000854	1.44	0.430870
D12	LAMA3	9.18	8.99	0.001719	0.001964	0.88	0.678911
E01	LAMB1	1.13	1.58	0.456631	0.334105	1.37	0.201049
E02	LAMB3	9.75	8.26	0.001163	0.003255	0.36	0.256610
E03	LAMC1	0.89	1.36	0.538577	0.389106	1.38	0.056254
E04	MMP1	-0.53	-0.02	1.4419	1.017202	1.42	0.238318
E05	MMP10	4.58	4.12	0.041799	0.057384	0.73	0.637562
E06	MMP11	7.68	7.11	0.00487	0.007256	0.67	0.424430
E07	MMP12	10.63	14.78	0.00063	0.000036	17.71	0.373191
E08	MMP13	16.48	16.23	0.000011	0.000013	0.84	0.628353
E09	MMP14	2.4	2.19	0.189256	0.218687	0.87	0.871463
E10	MMP15	12.49	13.47	0.000174	0.000088	1.98	0.231498
E11	MMP16	5.69	6.27	0.019349	0.012915	1.50	0.183187
E12	MMP2	1.11	0.97	0.462092	0.50978	0.91	0.573546
F01	MMP3	13.73	13.89	0.000074	0.000066	1.12	0.731364
F02	MMP7	10.01	9.54	0.000971	0.001345	0.72	0.818732
F03	MMP8	16.38	16.57	0.000012	0.00001	1.13	0.813477
F04	MMP9	16.32	15.66	0.000012	0.000019	0.63	0.402307
F05	NCAM1	13.29	15.91	0.0001	0.000016	6.17	0.046454
F06	PECAM1	3.31	3.93	0.100651	0.065422	1.54	0.621359
F07	SELE	5.1	5.97	0.029148	0.015907	1.83	0.402759
F08	SELL	9.08	7.93	0.001846	0.004089	0.45	0.074673
F09	SELP	7.77	6.92	0.004583	0.00828	0.55	0.205867
F10	SGCE	4.99	5.51	0.031477	0.021938	1.43	0.251018
F11	SPARC	-1.58	-0.56	2.994117	1.47936	2.02	0.001120
F12	SPG7	5.56	5.51	0.021163	0.021955	0.96	0.954220
G01	SPP1	9.38	10.76	0.001504	0.000578	2.60	0.302247
G02	TGFBI	4.75	5.62	0.037102	0.020382	1.82	0.287751
G03	THBS1	-3.9	-2.88	14.896099	7.376909	2.02	0.015251
G04	THBS2	10.27	11.16	0.000811	0.000437	1.85	0.406394
G05	THBS3	9.68	8.72	0.001221	0.002372	0.51	0.121761
G06	TIMP1	4.58	4.18	0.041804	0.055082	0.76	0.243837
G07	TIMP2	0.64	0.84	0.642928	0.559352	1.15	0.565337
G08	TIMP3	7.99	11.04	0.003943	0.000476	8.29	0.386948
G09	TNC	16.46	14.38	0.000011	0.000047	0.24	0.288267
G10	VCAM1	9.01	8.13	0.001942	0.00357	0.54	0.812977
G11	VCAN	14.3	11.39	0.000049	0.000373	0.13	0.242396
G12	VTN	11.24	13.16	0.000413	0.000109	3.77	0.325615
H01	ACTB	-2.91	-2.88	7.510101	7.375274	1.02	0.827371
H02	B2M	-0.27	-0.37	1.207778	1.288915	0.94	0.742064
H03	GAPDH	-1.44	-1.29	2.718774	2.444897	1.11	0.601017
H04	HPRT1	5.66	5.36	0.019748	0.024336	0.81	0.299149
H05	RPLP0	-1.04	-0.82	2.05343	1.767995	1.16	0.667373

Supplementary Table S3. Differential gene expression analysis of RT2 Profiler PCR Array Human Extracellular Matrix & Cell Adhesion Molecules Kit. HUVEC transfected with miR-133a-3p or negative control miR-NC were stimulated with VEGF-A165 for 4 hour. Upregulated and downregulated genes are highlighted in red and green, respectively. Genes showing values of AVG ΔCt ≥ 10 indicating high number of amplification cycles required in the PCR analysis were not considered in further experiments to improve accuracy and reproducibility of data.

GENES	miR-NC 0h									miR-133a-3p 0h								
PLAUR	0.64	1.49	1.05	1.04	0.94	1.03	1.02	0.96	1.02	0.29	0.35	0.38	0.26	0.30	0.31	0.51	0.59	0.59
MSN	0.91	1.26	0.87	0.98	0.97	1.05	0.85	1.27	0.92	0.51	0.70	0.50	0.58	0.65	0.62	0.59	0.61	0.59
RCAN1.4	1.01	0.91	1.09	0.94	1.10	0.97	0.99	0.98	1.04	2.06	1.81	2.37	1.17	1.39	1.38	1.33	1.46	1.45
CD44	1.00	1.31	0.76	0.91	1.00	1.09	1.06	0.95	0.99	0.18	0.34	0.21	0.24	0.21	0.20	0.47	0.33	0.50
ID1	0.79	2.47	0.51	1.12	0.94	0.95	1.71	0.35	1.69	0.19	0.30	0.22	0.38	0.34	0.37	0.70	0.76	0.58
CCNE1	0.75	0.95	1.41	0.85	1.08	1.09	0.78	1.25	1.03	0.86	1.07	1.06	0.63	0.70	0.69	0.64	0.71	0.68
CCND1	0.80	0.99	1.25	0.92	1.07	1.01	0.80	1.24	1.01	0.95	1.14	1.29	0.83	0.91	0.73	0.59	0.79	0.83
DLL4	1.02	0.84	1.17	0.71	1.11	1.26	0.90	1.13	0.98	8.28	8.05	13.28	9.04	11.88	10.08	8.16	7.74	6.74
HEY1	0.97	1.09	0.95	1.00	0.97	1.03	1.04	1.01	0.95	4.65	4.59	5.04	3.46	3.76	3.97	2.67	1.47	5.81
JAG2	0.69	1.29	1.13	0.70	1.01	1.40	0.73	1.23	1.11	3.69	4.07	3.96	3.53	3.41	3.41	2.81	3.30	2.82
NOTCH4	0.79	1.20	1.06	0.79	1.03	1.22	1.04	1.01	0.96	3.72	4.01	4.02	4.35	6.35	4.92	4.19	3.84	2.69
NRARP	0.87	1.04	1.10	0.79	0.94	1.34	0.82	1.09	1.12	3.48	3.59	3.09	2.98	3.66	4.23	2.99	2.52	2.65
HES4	0.83	1.24	0.97	0.92	0.94	1.15	0.75	1.11	1.19	4.84	5.74	5.02	4.10	5.33	4.79	4.59	4.82	3.27
TIMP3	1.15	0.78	1.12	0.95	1.02	1.03	1.26	0.81	0.98	10.98	10.9	12.24	5.156	5.643	4.86	5.92	6.38	4.59
ADAMTS1	0.99	1.41	0.72	nd	nd	nd	0.91	1.28	0.86	0.70	1.02	0.85	nd	nd	nd	0.66	0.49	0.73
ITGa6	1.09	0.82	1.11	0.99	1.03	0.97	0.90	1.40	1.12	2.07	2.29	2.31	2.03	1.84	2.06	1.59	2.41	1.59
THBS1	1.07	1.01	0.92	0.96	0.97	1.08	0.78	1.40	0.91	1.75	2.48	1.70	1.90	2.21	1.89	1.90	1.47	1.50
SPARC	0.86	0.98	1.19	0.74	1.19	1.13	0.80	1.07	1.17	2.79	2.64	3.45	2.76	2.67	2.65	2.25	2.34	2.25
CDK1	0.89	nd	1.13	0.85	0.98	1.20	0.76	1.08	1.22	0.22	0.26	0.22	0.19	0.19	0.22	0.22	0.23	0.28
CCNA2	0.89	nd	1.12	0.77	1.21	1.07	0.88	1.00	1.13	0.21	0.22	0.22	0.14	0.15	0.16	0.14	0.19	0.24
CCNB1	0.86	nd	1.16	0.83	1.04	1.16	0.95	1.01	1.05	0.27	0.30	0.25	0.18	0.22	0.23	0.20	0.26	0.28
MCM2	0.84	1.03	1.15	0.90	1.00	1.10	1.00	0.98	1.02	0.38	0.42	0.42	0.37	0.42	0.42	0.60	0.53	0.51
MCM4	0.80	1.07	1.17	0.80	1.11	1.13	0.85	0.89	1.32	0.43	0.47	0.40	0.35	0.43	0.38	0.35	0.40	0.58
CCND3	0.65	1.72	0.89	0.76	1.14	1.16	0.87	1.05	1.09	1.22	1.32	1.41	1.73	1.95	1.86	1.71	1.86	1.79
CCNG1	1.24	0.87	0.93	0.80	1.05	1.20	0.82	1.08	1.13	2.59	2.87	2.63	2.48	2.53	2.84	2.20	2.44	2.33

GENES	miR-NC VEGF 1h									miR-133a-3P VEGF 1h								
PLAUR	0.90	0.91	1.06	0.85	0.94	1.02	1.02	0.95	1.01	0.39	0.49	0.51	0.34	0.40	0.24	0.47	0.50	0.58
MSN	1.11	1.10	1.13	0.85	1.02	1.00	1.04	0.88	1.07	0.69	0.64	0.59	0.32	0.40	0.53	0.66	0.67	0.72
RCAN1.4	14.95	6.75	18.70	13.23	10.52	9.45	13.19	12.79	16.98	76.10	68.17	58.90	63.43	78.58	69.36	62.67	80.67	51.54
CD44	0.90	0.98	0.86	1.01	0.97	0.92	1.15	1.21	0.95	0.28	0.25	0.29	0.14	0.19	0.21	0.34	0.36	0.39
ID1	0.76	0.45	0.42	0.87	0.94	1.25	0.56	0.50	0.48	0.40	0.25	0.53	0.29	0.30	0.43	0.45	0.46	0.39
CCNE1	1.05	1.77	0.98	1.78	1.14	1.02	0.96	1.20	0.88	0.80	0.96	0.93	0.69	0.99	0.76	0.64	0.61	0.72
CCND1	1.11	1.84	0.99	nd	1.72	0.84	1.32	1.69	1.04	0.99	1.30	1.29	0.81	0.78	0.85	0.62	0.70	0.85
DLL4	1.76	1.95	1.96	1.30	2.69	1.74	2.45	3.58	2.40	14.57	17.91	15.95	11.73	21.50	21.22	14.39	14.08	16.60
HEY1	4.31	6.20	4.73	3.28	9.32	2.07	4.11	10.89	3.21	19.52	21.93	15.03	13.20	11.10	12.63	17.99	14.11	6.80
JAG2	1.01	1.76	0.91	1.12	1.96	0.87	0.96	1.66	1.30	3.48	3.23	3.36	3.58	3.02	4.18	2.72	2.48	2.69
NOTCH4	1.43	1.36	0.70	1.22	2.41	1.01	0.81	1.30	0.89	2.59	3.12	2.79	4.34	4.94	4.19	2.69	2.89	2.92
NRARP	1.17	2.25	1.18	1.60	3.31	1.30	2.35	3.39	2.73	4.40	4.58	4.80	4.85	5.02	6.68	5.03	4.47	5.59
HES4	0.91	1.62	0.88	1.19	2.18	0.91	1.18	1.85	0.84	4.97	4.03	4.60	4.18	4.57	5.60	4.48	3.77	4.18
TIMP3	0.702	0.576	0.733	0.7719	0.63	0.65	1.164	0.614	1.225	6.51	5.79	6.77	5.44	5.18	5.79	5.01	5.06	5.95
ADAMTS1	3.37	3.05	5.27	nd	nd	nd	3.06	3.15	3.24	9.64	8.71	8.31	nd	nd	nd	6.14	4.75	5.91
ITGa6	0.83	0.90	0.90	0.87	0.82	0.71	0.95	0.87	1.09	1.26	1.50	1.59	2.16	1.67	1.61	1.50	0.92	1.50
THBS1	1.18	1.17	1.17	0.97	1.01	0.94	1.28	1.34	1.27	2.25	1.87	2.17	1.53	1.93	2.66	1.89	2.12	2.15
SPARC	1.15	1.00	1.07	0.88	1.41	0.65	1.09	1.57	1.35	2.21	2.18	2.4	2.08	1.78	2.27	2.24	2.56	2.21
CDK1	1.06	1.93	1.04	1.20	1.55	0.64	1.13	2.15	0.96	0.25	0.23	0.30	0.17	0.16	0.20	0.22	0.25	0.28
CCNA2	1.12	1.98	1.09	1.73	1.08	1.09	0.74	1.34	0.65	0.20	0.23	0.27	0.13	0.18	0.13	0.12	0.11	0.15
CCNB1	1.15	2.05	0.93	1.04	1.59	1.01	1.11	1.44	0.82	0.29	0.25	0.32	0.18	0.14	0.19	0.19	0.17	0.26
MCM2	1.01	1.49	0.99	1.05	1.46	0.78	1.14	1.89	1.16	0.36	0.37	0.44	0.49	0.43	0.50	0.36	0.39	0.47
MCM4	1.21	1.83	0.92	1.03	1.69	0.91	0.88	1.18	0.76	0.50	0.48	0.30	0.37	0.33	0.40	0.29	0.25	0.35
CCND3	0.73	1.15	0.64	1.24	1.98	1.05	0.69	1.01	0.55	1.00	1.03	1.09	1.73	1.76	2.02	0.86	0.85	0.90
CCNG1	1.23	1.98	1.07	0.99	1.58	0.97	0.74	1.38	0.87	2.49	2.51	2.62	1.71	2.31	2.10	1.89	1.60	1.81

Supplementary Table S4. Differential gene expression of angiogenic-related genes in HUVEC expressing miR-133a-3p or miR-NC. HUVEC transfected with miR-133a-3p (blue columns) or negative control miR-NC (grey columns) were left unstimulated (0h) or were stimulated with VEGF-A165 for 1 hour. Rows show fold-induction RNA levels for each gene referred to miR-NC 0h. Ct values were normalised using the Ct values for the housekeeping gene *Hprt-1*. Analysis of data was carried out using the comparative $2^{-\Delta\Delta Ct}$ method. nd = non-determined.

Genes	miR-133a-3p miRWalk	miR-133a-3p TargetScan	miR-133a-3p miRDB	miR-133a-5p miRWalk	miR-133a-5p TargetScan	miR-133a-5p miRDB
VEGF-A	YES/5UTR	NO	NO	NO	NO	NO
VEGF-B	NO	NO	NO	YES	YES	NO
VEGF-C	NO	NO	NO	NO	NO	NO
VEGFR1	NO	NO	NO	NO	NO	NO
VEGFR2	NO	NO	NO	NO	NO	NO
VEGFR3	NO	NO	NO	NO	NO	NO
CALCINEURIN-A	YES	NO	NO	NO	NO	NO
CALCINEURIN-B	YES	NO	NO	NO	NO	NO
NFAT1	NO	NO	NO	NO	NO	NO
NFAT2	NO	NO	NO	NO	NO	NO
NFAT3	YES	NO	NO	YES	NO	NO
NFAT4	YES	NO	NO	NO	NO	NO
NFAT5	YES	YES	YES	YES	YES	YES
JNK	NO	NO	NO	YES	NO	NO
p38	NO	NO	NO	NO	NO	NO
ERK	NO	NO	NO	NO	NO	NO

Supplementary Table S5. Bioinformatic analysis of miR-133a predicted binding sites in candidate genes encoding well-known mediators of VEGF-induced angiogenesis. The sequences of the indicated genes were analysed for potential binding sites for miR-133a-3p, or miR-133a-5p using miRWalk, Targetscan, and miRDB data bases.

Genes	miR-133a-3p miRWalk	miR-133a-3p TargetScan	miR-133a-3p miRDB	miR-133a-5p miRWalk	miR-133a-5p TargetScan	miR-133a-5p miRDB
PLAUR	YES	NO	NO	NO	NO	NO
MSN	NO	YES	YES	NO	NO	NO
RCAN1.4	YES	NO	NO	YES	YES	NO
CD44	YES	NO	NO	YES	YES	NO
ID1	NO	NO	NO	NO	NO	NO
CCNE1	YES	NO	NO	YES	NO	NO
CCND1	YES	NO	NO	NO	NO	NO
DLL4	YES	NO	NO	YES	NO	NO
HEY1	YES	NO	NO	YES	YES	NO
JAG2	YES	NO	NO	YES	NO	NO
NOTCH4	YES	NO	NO	YES	NO	NO
NRARP	NO	NO	NO	NO	NO	NO
HES4	YES	NO	NO	NO	YES	NO
TIMP3	YES	NO	NO	YES	NO	NO
ADAMTS1	YES	NO	NO	YES	YES	NO
ITGA6	NO	NO	NO	YES	NO	NO
THBS1	YES	NO	NO	YES	NO	NO
SPARC	YES	NO	NO	NO	NO	NO
CDK1	NO	NO	NO	NO	YES	NO
CCNA2	NO	NO	NO	NO	NO	NO
CCNB1	YES	NO	NO	NO	NO	NO
MCM2	NO	NO	NO	YES	NO	NO
MCM4	NO	NO	NO	NO	NO	NO
CCND3	YES	NO	NO	NO	YES	YES
CCNG1	NO	NO	NO	NO	NO	NO

Supplementary Table S6. Bioinformatic analysis of miR-133a predicted binding sites in the differentially expressed group of genes described in this study. The sequences of the indicated genes were analysed for potential binding sites for miR-133a-3p, or miR-133a-5p using miRWalk, Targetscan, and miRDB data bases. None of the genes was predicted to contain miR-133a binding sites by all the data bases used.

Genes	Taqman Assay ID
MSN	Hs00741306_mH
PLAUR	Hs00958880_m1
CD44	Hs01075864_m1
ID1	Hs03676575_s1
CCNE1	Hs01026536_m1
CCND1	Hs00765553_m1
DLL4	Hs00184092_m1
HEY1	Hs05047713_s1
JAG2	Hs00171432_m1
NOTCH4	Hs00965889_m1
NRARP	Hs04183811_s1
HES4	Hs00368353_g1
TIMP3	Hs00165949_m1
ITGA6	Hs01041011_m1
ADAMTS1	Hs00199608_m1
THBS1	Hs00962908_m1
SPARC	Hs00234160_m1
CDK1	Hs00938777_m1
CCNA2	Hs00996788_m1
CCNB1	Hs01030099_m1
MCM2	Hs01091564_m1
MCM4	Hs00907398_m1
CCND3	Hs01017690_g1
CCNG1	Hs00171112_m1
RCAN1.4	Hs01120957_m1
HPRT1	Hs99999909_m1

Supplementary Table S7. References of TaqMan Gene Expression Assays (Applied Biosystems) used in this work.
